# Recent Advances in Marine-Based Nutraceuticals and Their Health Benefits

**DOI:** 10.3390/md18120627

**Published:** 2020-12-09

**Authors:** Vida Šimat, Nariman Elabed, Piotr Kulawik, Zafer Ceylan, Ewelina Jamroz, Hatice Yazgan, Martina Čagalj, Joe M. Regenstein, Fatih Özogul

**Affiliations:** 1 University Department of Marine Studies, University of Split, Ruđera Boškovića 37, 21000 Split, Croatia; mcagalj@unist.hr; 2 Laboratory of Protein Engineering and Bioactive Molecules (LIP-MB), National Institute of Applied Sciences and Technology (INSAT), University of Carthage, Avenue de la République, BP 77-1054 Amilcar, Tunisia; elabed_nariman@yahoo.fr; 3 Department of Animal Products Technology, Faculty of Food Technology, University of Agriculture in Cracow, ul. Balicka 122, 30-149 Krakow, Poland; piotr.kulawik@urk.edu.pl; 4 Department of Gastronomy and Culinary Arts, Faculty of Tourism, Van Yüzüncü Yıl University, 65080 Van, Turkey; zaferceylan@yyu.edu.tr; 5 Institute of Chemistry, Faculty of Food Technology, University of Agriculture in Cracow, ul. Balicka 122, 30-149 Krakow, Poland; ewelina.jamroz@urk.edu.pl; 6 Faculty of Veterinary Medicine, Cukurova University, 01330 Adana, Turkey; hyazgan@cu.edu.tr; 7 Department of Food Science, Cornell University, Ithaca, NY 14853-7201, USA; jmr9@cornell.edu; 8 Department of Seafood Processing Technology, Faculty of Fisheries, Cukurova University, 01330 Adana, Turkey

**Keywords:** nutraceuticals, pharmaceuticals, bioactive compounds, functional foods, marine resources

## Abstract

The oceans have been the Earth’s most valuable source of food. They have now also become a valuable and versatile source of bioactive compounds. The significance of marine organisms as a natural source of new substances that may contribute to the food sector and the overall health of humans are expanding. This review is an update on the recent studies of functional seafood compounds (chitin and chitosan, pigments from algae, fish lipids and omega-3 fatty acids, essential amino acids and bioactive proteins/peptides, polysaccharides, phenolic compounds, and minerals) focusing on their potential use as nutraceuticals and health benefits.

## 1. Introduction

The raw foods, functional foods or dietary supplements that contain bioactive molecules and have ability to provide health benefits (prevention and treatment of disease) beyond their nutritional value are known as “nutraceuticals” [1]. This term combines two words, nutrient and medicinal component (pharmaceutical). In recent years, functional and bioactive compounds from natural sources such as terrestrial and marine plants, animals, or even microorganisms have become sustainable solution that offers new molecules with strong biological activity. With the increase in the field of health-based research these new molecules are gaining more importance. Modern dietary habits and lifestyle in developed countries have resulted in an increasing number of diseases such as type 2 diabetes mellitus, obesity, metabolic syndrome, cancer, or neurodegenerative diseases [2]. Bioactive components from natural sources with an ability to contribute to the overall health have become an interesting alternative to potentially harmful synthetic ingredients.

The high diversity and dynamics of marine ecosystem makes an ideal reservoir identification of new molecules and development of marine nutraceuticals. More than 20,000 marine bioactive compounds have been isolated, however, only a small proportion of them have been thoroughly studied and exploited to some degree [3]. Marine ecosystems have earned a title “Natural Medicine Chest of the New Millennium” and are becoming an important market worldwide. In 2018, the global market for marine-derived compounds was over 10 billion US dollars, which is expected to rise to $22 billion by 2025 at a compound annual growth rate of 11.3% from 2019–2025 [4]. The compounds from marine sources that have shown beneficial health effects and a potential use in food and medical applications, include protein and peptides, polysaccharides, ω-3 polyunsaturated fatty acids (PUFA), enzymes, polyphenolic compounds, pigments, and vitamins [5,6,7]. 

Generally, synthetic chemical drugs are still used in medical practice to treat diverse acute or chronic diseases, mainly various disorders involving the immune system’s chronic inflammatory states, allergic reactions, diabetes, cardiovascular diseases, severe human tumors, and cancers or as immunosuppressant during transplantation. Despite their treatment’s effectiveness, there are often significant human health concerns regarding the side effects of many of these synthetic chemical drugs and conventional antibiotics due to the misuse, uncontrolled application, and overuse of these compounds and sometimes even to their proper use. The application and especially misuse of some synthetic antimicrobial drugs may be among the principal factor responsible for the development of antibiotic-resistant microbes (ARM). The power of natural nutraceuticals is in their ability to improve the quality of life, prevent or even treat some conditions without any adverse side effects. The safe nature of marine nutraceuticals makes them more desirable, therefore, it may be beneficial to develop non-toxic but effective natural agents as alternatives to the chemical compounds [6]. Many marine organisms, including fish, crustaceans, algae, molluscs, sponges, actinomycetes, fungi and microorganisms have been exploited as sources of natural components [6,7]. 

This review paper provides an overview of diverse marine-based beneficial compounds with great potential as nutraceuticals or application in the food industry that have been in the focus of research in recent years. These include chitin, chitosan, pigments, polysaccharides, and polyphenols from algae, fish oil, fatty acids (FA), essential amino acids (EAA), peptides, gelatin, vitamins, minerals, and dopamine. Not all of them are bioactive compounds *sensu stricto* but they have positive impact on human health because of their beneficial nutritional properties. Many of them have strong biological properties but are prone to deterioration, thus nanotechnology was also reported as a way to protect these compounds and enhance their bioavailability after their application.

## 2. Marine-Based Beneficial Molecules

### 2.1. Chitin and Chitosan

Chitin is a naturally occurring polymer with crystalline forms (α, β, γ). α-Chitin is the most stable form of this polymer because it has an anti-parallel orientation of polysaccharide chains [8]. Chitosan ((1-4)-2-amino-2-deoxy-β-D-glucan) is the most common and natural cationic polysaccharide found in exoskeletons of crustaceans, mollusks, insects, and fungi. It is a product of chitin deacetylation, a process that removes acetyl groups (CH_3_–CO) from the molecule, which makes the biopolymer soluble in most dilute acids. In the deacetylation process, the amine (NH) groups are released, thus providing chitosan with cationic properties [8]. The main sources of chitosan production are by-products of seafood processing, such as crab shells and shrimp/prawn exoskeletons. 

**Figure 1:**
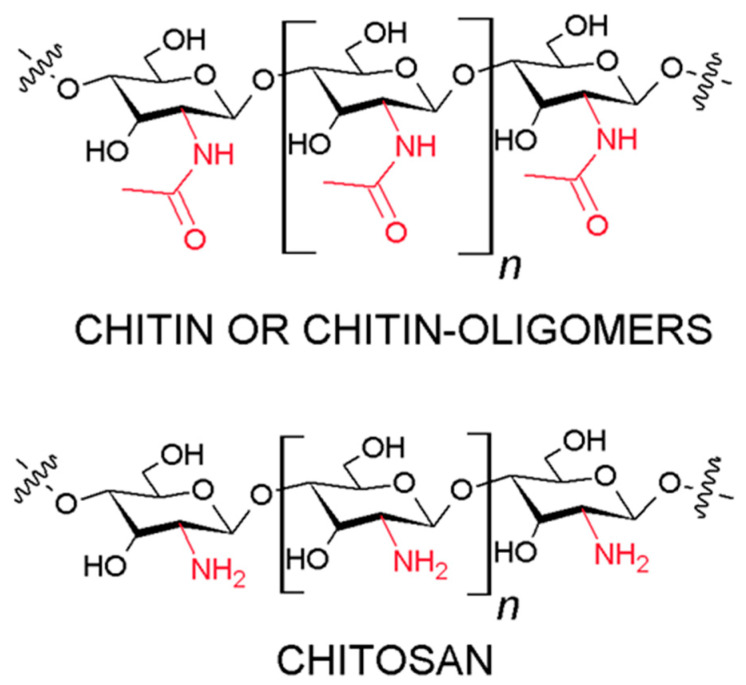
Structure of chitin and chitosan.

Chitin and chitosan are natural polymers with the same chemical structure (Figure 1). Both consist of a mixture of mainly N-acetyl-D-glucosamine and a small amount of D-glucosamine. Chitin is insoluble in an aqueous environment, while chitosan is soluble in an acidic environment due to the presence of free protonable amino groups present in the D-glucosamine units [9]. Chitooligomers (COS) are the degraded products of chitosan or chitin, which are obtained by enzymatic or chemical hydrolysis of chitosan. Chitosan has three types of reactive functional groups, an anamine/acetamide, as well as both primary and secondary hydroxyl groups in positions C-2, C-3, and C-6, respectively (Figure 1). Amine contents are the main factor influencing the differences in their structure and physicochemical properties.

The seafood processing industry generates large amounts of by-products and wastes (shells, scales, tails, heads, and guts) that can be a good source of functional ingredients. An example is shell material, which is a valuable source of chitin and chitosan. In this section, the health-promoting properties of chitin and chitosan, as well as their derivatives, are discussed.

#### 2.1.1. Antioxidant Properties

Oxidative stress can cause unanticipated enzyme activation and oxidative damage to cellular macromolecules, leading to a range of health disorders, including many cardiovascular diseases, inflammation, diabetes, neurodegenerative diseases and cancer. Antioxidants, including chitosan, and its derivatives, prevent oxidative damage by breaking the radical oxidation chain reaction [10]. 

The antioxidant effect of a dietary supplement (high molecular weight (MW) chitosan, trade name: Chitosamin^®^, ~100 kDa, 90% degree of deacetylation (DD)) was tested in healthy individuals. The preparation caused a decrease of the lipid hydroperoxides and uremic toxins in the gastrointestinal tract, which contributed to the inhibition of the subsequent development of oxidative stress in the human systemic circulation. Chitosamin^®^ can be used as a preparation in the antioxidant treatment of many diseases, e.g., in renal failure [11].

The antioxidant activity of chitosan depends on its MW, DD, and source of origin. Je et al. [12] tested three varieties of chitosan with different MW (5–10, 1–5, and <1 kDa) from partially deacetylated chitosan preparations (90%, 75%, and 50%). The best O_2−_, OH and DPPH capturing could be observed with the 1–5 kDa chitosan preparation containing 90% deacetylated chito-oligosaccharides. Similar results were obtained by Anraku et al. [13], where high (HMWC; 1000 kDa) and low MW (LMWC; 30 kDa) chitosan were used to study its effect on oxidative stress in normal and metabolic syndrome model rats. High antioxidant activity was observed in rats where LMWC was used in the diet. On the other hand, diets with HMWC decreased the levels of pro-oxidants such as low lipoprotein cholesterol (LDL) in the gastrointestinal tract, thus inhibiting the subsequent development of oxidative stress in the systemic circulation.

Goto et al. [14] investigated the protective and antioxidant effects of surface-deacetylated chitin nanofibers (SDACNF) on the liver in rats. Administration of SDACNF (80 mg/kg/day) for 8 weeks of reduced liver damage and oxidative stress compared to untreated rats.

#### 2.1.2. Antimicrobial Properties

The potential mechanism of chitosan and chitin antimicrobial action is based on the polycationic nature of the biopolymer (i.e., the presence of NH_3_^+^ groups) which interact with negatively charged surface components of many microorganisms, in turn leading to leakage of cellular substances, and subsequent, cell death [15]. Both lipopolysaccharides in Gram-negative bacteria and teichoic acid in Gram-positive bacteria have an important role in the interaction with chitosan [16]. The greater the number of positively charged amino groups in the structure of a biopolymer, the greater its antimicrobial activity. Chitosan contains a greater number of positively charged amino groups than chitin, and therefore, has higher antimicrobial activity [17]. The antimicrobial activity of chitosan and chitin is influenced by many different factors, i.e., MW, degree of polymerization, pH, and DD [15]. Moreover, the source of chitosan and chitin origin influences their antimicrobial properties. Chien et al. [18] reported that raw chitin from crab shells did not show any antibacterial activity, but after the purification process, it showed activity against *Escherichia coli*. Chitin from shiitake stipes showed better antimicrobial activity against pathogens than chitin from crab shell. The process of chitin discoloration could have an impact on its antimicrobial activity. After cleaning, chitosan from shiitake stipes and crab shells showed good antimicrobial activity against eight pathogens. However, chitosan from shiitake stipes was more effective than from crab shells.

Chitosan is poorly soluble in organic solvents, which may limit its potential use. To adjust the physicochemical properties for specific applications, chitosan’s amino and hydroxyl groups can undergo various modifications. Amine groups readily react with aldehydes and ketones to form Schiff bases. Hamed et al. [19] obtained three new Shiff base chitosan derivatives with reactions with a pyrazole heterocycle compound. The chitosan derivatives showed strong antimicrobial activity against *E. coli* and *Klebsiella* as Gram-negative bacteria, *Staphylococcus aureus*, and *Streptococcus mutans* as Gram-positive bacteria as well as *Aspergillus fumigatus*, and *Candida albicans* as fungi. Moreover, the MMT test did not show any cytotoxic activity against normal retinal cells. N-selective chitosan derivatives were obtained: N-methylchitosan (NMC), trimethylchitosan (TMC), diethylmethyl chitosan (DEMC), and carboxymethyl chitosan (CMC). The replacement of the alkyl groups enhanced the antimicrobial activity of chitosan. However, TMC showed the best antimicrobial response against *E. coli* and *S. aureus*, which may be due to the presence of positive charges on the chitosan skeleton. The results indicate that the quaternary derivative, O-methyl free N,N,N-trimethyl chitosan (TMC), had the best antimicrobial properties and good biocompatibility [20]. Salama et al. [21] synthesized chitosan derivatives having guanidinium functions. Of the four derivatives, N-guanidinium chitosan acetate showed the best antimicrobial activity against *E. coli*, *P. aeruginosa*, *S. aureus*, *B. subtilis*, and *C. albicans*, and they reported low minimal inhibitory concentration (MIC) values for all the microorganisms.

#### 2.1.3. Anti-Hypertensive Activity

Hypertension causes the development of cardiovascular disease (CVD). In the human blood, angiotensin-I converting enzyme (ACE) contributes to the regulation of blood pressure by converting inactive angiotensin I into its active form, angiotensin II, and this causes small blood vessels to narrow and blood pressure to rise. To prevent hypertension, inhibition of ACE activity may be beneficial [10]. Chitosan derivatives—COS, in particular, showed antihypertensive effects. Their inhibitory effect on ACE is dependent on the DD and MW of the compound [22]. Huang et al. [23] modified COS with -COCH_2_CH_2_COO- groups. CeCOS strongly inhibited ACE and its activity was comparable to that of Captopril. In addition to ACE, renin also has a significant role in the renin-angiotensin system (RAS).

Renin cleaves plasma angiotensinogen to angiotensin-I, which is further converted by ACE to angiotensin-II. Renin inhibition is a potential antihypertensive strategy. Park et al. [24] prepared 6 types of COS with different MW (10–5, 5–1, and <1 kDa) and DD (90% and 50%). The results indicated that both DD and MW have an influence on renin inhibitory activity. Deacetylated COS (90%) showed a higher renin inhibitory activity than 50% deacetylated COS.

#### 2.1.4. Anti-Allergy and Anti-Inflammatory Activity

Allergies are caused by an interaction between an antigen and the antigen-specific IgE. On the other hand, asthma is an allergic disease characterized by increased respiratory tract responsiveness. Vo et al. [25] showed that with COS with three different MW ranges (1–3, 3–5 and 5–10 kDa), the lowest MW attenuated allergic reactions by inhibiting degranulation and cytokine production in mast cells. Chung et al. [26] investigated the anti-inflammatory effect of LWM COS prepared from HMW chitosan as a result of enzymatic digestion against allergic reactions and allergic asthma in vivo and in vitro. The results indicated that LMW-COS had anti-inflammatory effects related to the regulation of Th2 and proinflammatory cytokines and therefore, may be a promising candidate for the development of a potent therapeutic agent for the treatment of allergic asthma.

#### 2.1.5. Anti-Obesity and Anti-Diabetic Activity

The increase in the number of people with obesity is becoming a global burden on public health. In epidemiological studies, it has been shown that a lower incidence of obesity-related diseases has been observed in populations where seafood is consumed. Certain ingredients in seafood are believed to have a positive effect on the fight against obesity [27]. Chitosan and its derivatives are used in the treatment of obesity-related diseases [27,28,29]. The use of chitosan in dietary supplementation effectively reduces the level of total cholesterol (TC) and LDL-C in the plasma, and the level of triacylglycerol (TG) in the liver as well as plasma. Lowering the level of lipids in the plasma results from the ability of chitosan to bind dietary lipids and bile acids, and inhibit the activity of pancreatic lipase, thus reducing the absorption of intestinal fat in the gastrointestinal tract [22].

High DD and MW chitosan has higher fat binding capacity than low DD and MW chitosan [30]. Chitosan in tablet form has been shown to be a safe dietary supplement, benefiting human health. The mechanism of action of hypolipidemic chitosan is attributed to its ability to bind to fats, cholesterol, and bile salts. Hydrophobic interactions and hydrogen bonds between chitosan and lipids as well as the electrostatic attraction between positively charged amino groups of chitosan and negatively charged carboxyl groups of FA and bile salts are the cause of chitosan’s hypolipidemic effects [30,31,32]. Azuma et al. [33] applied surface-deacetylated chitin nanofibers (SDCH-NF) in the diets of rats. The oral administration of low molecular weight chitosan increased the levels of ATP and 5-HT in the plasma by activating the intestinal microflora. Their results suggested that the anti-obesity effect of SDCH-NF might be due to changes in the gut microflora population. There are studies in which weight gain reduction in overweight subjects has been confirmed [33,34,35]. However, it was also stated that chitosan/chitin had only a minor effect on weight loss and is unlikely to be of clinical relevance [36].

A recent study investigated the effect of low molecular weight chitosan in the diet of mice with type-1 diabetes. Daily administration of chitosan in the drinking water (0.8%) reduced the levels of serum glucose, urine glucose, and serum triglycerides in the mice leading to a decrease in hyperglycemia, hypertriglyceridemia, polydipsia, and polyuria among the tested animals [37].

#### 2.1.6. Anti-Cancer and Anti-Tumor Activity

Chitosan also has an anti-cancer effect by limiting the growth of cancer cells. The anti-tumor activity results from the potential stimulating effect on the immune system [9] as well as inhibiting angiogenesis and apoptosis from DNA fragmentation [10]. Chitosan (500 kDa, 70% DD) inhibited the activity of MMP-2 melanoma cells. Although the expression level of MMP-2 was not altered, the amount of MMP-2 in the cell supernatant was reduced. This behavior can be attributed to the post-transcriptional effect of chitosan on MMP-2. Direct molecular interaction between MMP-2 (using atomic force microscopy) and chitosan was observed, as well as non-competitive inhibition of MMP-2 by chitosan (using a colorimetric test) [38].

Sayari et al. [39] extracted chitin from the by-products of *N. norvegicus*, and then chitosan was obtained by partial deacetylation of chitin. The biopolymer showed antiproliferative activity against HCT116 human colon cancer cells. HCT116 cell proliferation was significantly inhibited between 13.5 and 67.5% at 0.5–6 mg/mL chitosan after 24 h of cell treatment.

Resmi et al. [40] extracted chitosan nanoparticles from shrimp shell waste using two successive steps: demineralization and deproteinization. Compared to chemically synthesized chitosan, chitosan NP showed an inhibitory effect on the proliferation of MCF-7 breast cancer cells and minimal cytotoxicity of normal L929 fibroblast cells. El-Naggar et al. [41] used freshwater crayfish waste from *Procambarus clarkii* as a precursor to obtaining chitin, which was deacetylated to obtain chitosan. The chitosan was transformed into chitosan NP and Schiff bases. Cytotoxic activity against three cell lines (HepG-2, HCT-116 and MCF-7) indicated the best anti-tumor activity for chitosan Schiff bases, followed by chitosan NP. Chitosan showed the lowest anti-tumor activity of all the tested compounds. Sedghi et al. [42] prepared nanofibers of chitosan derivatives. The results of the MMT test indicated that the material had good activity against 4T1 breast cancer cells and did not show any cytotoxic effects on normal cells, suggesting a promising application of nanofibers in the prevention of breast cancer recurrence.

### 2.2. Beneficial Molecules from Marine Macroalgae

#### 2.2.1. Pigments

**Figure 2:**
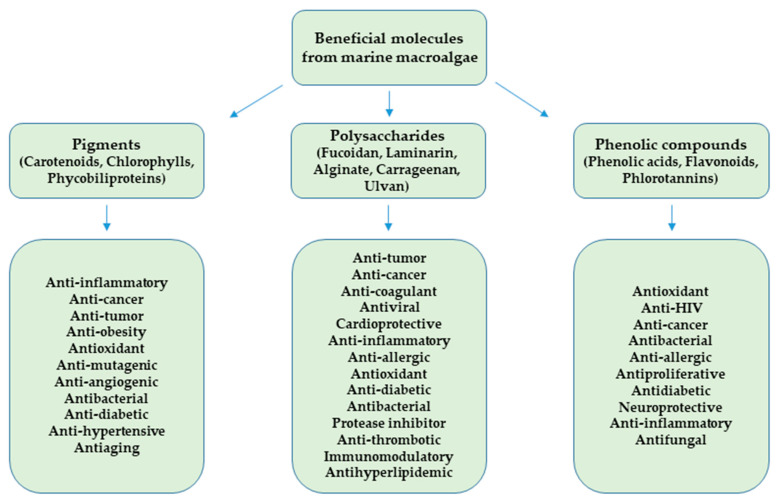
Biological properties of beneficial molecules derived from marine macroalgae.

Among the biological species, marine algae are classified as one of the primitive producers of whole aquatic biomass making them a sources of natural bioactive molecules [43,44,45,46]. Several functional metabolites derived from marine algae have shown health benefits [47]. The natural algal pigments (NPM) are often bioactive, particularly from Chlorophyceae, Rhodophyceae, and Phaeophyceae (or green, red, and brown algae, respectively) [43,47]. The NPM synthesized by marine algae can be classified into three essential categories: carotenoids, chlorophylls, and phycobiliproteins [43]. Their stability is dependent on their chemical structures, which can be influenced by several factors, such as oxygen, light, heat, air, and pH [48]. The NPM are also characterized by their photosynthetic roles [47]. NMP have been applied in the food, nutraceuticals, and cosmetics industries [43] due to their antioxidant, antimicrobial, antidiabetic, antimalarial, anticancer, antiviral, anti-inflammatory, and anti-obesity activities (Figure 2) [44,49,50].

Carotenoids are an important class of fat-soluble NPM found in algae biomass and belong to the tetraterpenoids group consisting of a linear polyene chain (C40) [7,47,51]. Based on their chemical structure the carotenoids can be classified as unsaturated hydrocarbon carotenoids, commonly known as carotenes, including lycopene α-carotene and β-carotene pigments and xanthophylls, known as oxygenated carotenoids, such as astaxanthin, lutein, canthaxanthin, β-cryptoxanthin, and zeaxanthin [51,52]. Generally, fucoxanthin and astaxanthin are the most abundant and natural carotenoid pigments produced in seaweeds or marine algae [49]. On the other hand, the stability of carotenoid pigments is mainly affected by the influence of various environmental conditions, which can generate structural modifications due to oxidation, dehydrogenation, and hydrogenation reactions [53]. These NPM are considered to be antioxidants due to their role in the protection of the cells, retinal epithelium and skin against oxidative damages and thus reducing the risk of CVD, atherosclerosis, neurological and other non-communicable diseases (NCD) [7]. In particular, a fucoxanthin-rich fraction extracted from brown algae, *Sargassum siliquosum* and *S. polycystum*, was shown to have inhibitory activities on ACE, α-amylase and α-glucosidase, which potentially reduces CVD related risk [54]. Lutein and zeaxanthin extracted from macroalgae protect against macular degeneration [55]. Furthermore, the carotenoid pigments and particularly fucoxanthin can act as antitumor, anti-inflammatory, anticancer, anti-obesity and neuroprotective agents, and can treat rheumatoid arthritis, osteoporosis and diabetic disorders [47].

The other important algal pigments are the phycobiliproteins, which are water-soluble proteins and highly fluorescent compounds synthesized by blue-green and red algae [7,47]. These NPM are classified into the three categories of phycoerythrins, allophycocyanins, and phycocyanins [47]. These photosynthetic pigments have various significant pharmaceutical and health-improving applications, such as their use in histochemistry, immunoassays, flow cytometry, and cell imaging. They can be used for reactive oxygen substances (ROS) detection based on the antioxidant properties when isolated from *Porphyra* sp. [47]. Phycobiliproteins from macroalgae can also be used as natural colorings in chewing gums, dairy products, cosmetics and other products [56].

Another basic NPM isolated from marine algae is chlorophyll, which is a tetrapyrrole and greenish lipid soluble pigment. Generally, there are four major categories of chlorophylls found in marine algae: chlorophyll a, b, c, and d [47,48]. Chlorophyll pigments and their derivatives have cancer protective and anti-mutagenic effects [47] and may be taken into consideration for replacing the synthetic pigments that are used in the food industry [57].

#### 2.2.2. Polysaccharides 

Polysaccharides are sugar polymers, a biological macromolecule with different degrees of sulfation found in many species of marine algae [5], but also crabs and krill [58]. The polysaccharides isolated from marine organisms are considered safer and less costly than mammalian polysaccharides, thus more suitable for application in drug development, cosmetics, and functional food products [59]. Alginates, carrageenan, fucoidan, agar, furcelleran, ascophyllan, laminarin, polyuronides carrageenan, agar-agar, fucans, fucanoids, chitin and chitosan are marine-origin bioactive polysaccharides with anti-tumor, anti-coagulant, anti-virus, cardioprotective, anti-inflammatory, anti-allergic, anti-oxidant, anti-diabetic, antibacterial, and protease inhibitor activities [5,58,59]. Marine polysaccharides have antioxidant capacity through their scavenging of ROS [1].

The composition of polysaccharides in marine algae, the most important source of non-animal sulfated polysaccharides, varies according to the season, species, and geographic location [60]. The major sulfated polysaccharides found in brown algae include fucoidan, laminarin, and alginate, while carrageenan and ulvan are found in red and green algae, respectively.

Fucoidan is a sulfated polysaccharide found in the cell wall of brown macroalgae that has been studied for its biological activities [61]. Fucoidan’s structural complexity ranges from the basic structure that contains sulfate groups attached to fucose units to macromolecules containing a range of different monosaccharide units such as mannose, galactose, glucose, and xylose. This complexity depends on various factors such as species, harvesting time, and environmental conditions [62]. Fucoidan extracts have regulatory approvals for use in foods and dietary supplements for consumption up to 250 mg/day [63]. Anticancer effects of fucoidans on different cancer cell lines have been reported including inducing apoptosis in 5637 human bladder cancer cells [64], in human breast (MCF-7) and colon cancer cell (HCT15) lines [65], and the colon cancer adenocarcinoma (Caco-2) cell line [66]. Fucoidans from brown algae also have an inhibitory role in colony formation in human melanoma and colon cancer cells [67].

Laminarin is a brown algal polysaccharide with a low MW (~5 kDa). It is found in *Laminaria* and *Saccharina* species and to some extent in *Ascophyllum* and *Fucus* species. Laminarin is a storage β-glucan composed of (1,3)-β-D-glucan and some β-(1,6)-intrachain links [61,68]. Recently, laminarin’s anti-cancer effects were reported, including enhanced apoptotic cellular death, angiogenic potential inhibition, and colony formation inhibition [69]. Other than anticancer effects, laminarin from *Cystoseira barbata* (5% cream) significantly enhanced the in vivo healing process, improved wound contraction, accelerated re-epithelization, and allowed restitution of mice skin tissue [70]. Also, photo-cross-linkable laminarin-based hydrogels were developed for cell encapsulation and/or drug delivery [71].

Alginate is a linear polysaccharide consisting of (1-4)-linked β-D-mannuronic acid (M) and α-L-guluronic acid (G) monomers, constituting M-, G-, and MG- sequential block structures [72]. It is naturally present in the brown macroalgae cell wall. Alginates are being used in various applications, such as alginate fiber wound dressings, as excipients in drug delivery, as dental impression materials, and preventing gastric reflux [73]. Alginates are also used for food protection, as gelling, thickening, coating, emulsifying, and stabilizing agents in food products [74,75].

Carrageenan is sulfated polysaccharide with high molecular weight that consists of alternating linear chains of α-1,3-galactose and β-1,4,3,6-anhydrogalactose with ester sulfates (15–40%), and it is structural component of red macroalgae cell membranes [76]. It has various biological activities such as anti-thrombotic, anticancer, anti-viral, and immunomodulatory [77]. Due to its gelling mechanism and physiochemical properties, carrageenan is applied in drug delivery, bone and cartilage tissue regeneration and wound healing [78].

Ulvan, a water-soluble polysaccharide, is found in green macroalgae of the order Ulvales (*Ulva* and *Enteromorpha sp.*) [79]. Its main constituents are sulfate, rhamnose, xylose, iduronic and glucuronic acids [80]. Ulvan has shown various biological activities, including antioxidant, antiviral, anticancer, immunomodulating and antihyperlipidemic, and it also has the capacity to modulate cellular signaling processes in plant and animal systems which lead to beneficial effects on productivity and health [81]. Ulvan has also been reported to reduce total serum cholesterol, low density lipoprotein (LDL) cholesterol and triglycerides while elevating high density lipoprotein (HDL) cholesterol levels [82]. Abd-Ellatef et al. [83] tested ulvan using in vitro bioassays on a human breast cancer cell line (MCF-7) and an in vivo animal model of breast carcinogenesis and found that it had a potential chemo-preventive effect.

#### 2.2.3. Phenolic Compounds

Phenolics can be defined as substances with an aromatic ring having one or more hydroxyl groups, including their functional derivatives. Plants contain a large variety of phenolic derivatives including simple phenols, benzoic acid derivatives, phenylpropanoids, flavonoids, tannins, stilbenes, lignans, and lignins [84]. The phenolics from terrestrial sources have been well studied, but information on aquatic species is limited [2]. The natural production of phenolic compounds in marine organisms is related to environmental factors, such as salinity, UV radiation, nutrient availability, and temperature [46]. Marine macroalgae phenolics vary from simple molecules such as phenolic acids to highly complex compounds such as phlorotannins (PHT). Some phenolic compounds, such as bromophenols and PHT have been reported exclusively in marine sources [2,46]. PHT, assembled through polymerization of phloroglucinol units, are found in high amounts in brown algae [55]. They can be found in the cell wall or dissolved in cytoplasm and cell organelles [46]. The high reactivity PHT, particularly against oxidation, has increased the interest for food preservation and antiaging products, but also high-value commercial products within the pharmaceutical, cosmeceutical, nutraceutical, and food industries [85]. Phenolics from macroalgae are considered natural anti-allergic compounds for allergy remission [86], as functional ingredients in pharmaceuticals and foods for the treatment and/or prevention of neurodegenerative disease and CVD [87,88], and as compounds that have potential antidiabetic effects through the inhibition of both α-amylase and α-glucosidase [89].

Besides pigments, polysaccharides, and phenolics, marine macroalgae also contain other beneficial molecules such as fatty acids, proteins and vitamins. Various researchers did in vivo studies on the beneficial effects of algae ingestion in rats. For example, Liu et al. [90] reported that supplementation of *Gelidium amansii* in the diet can improve the increased insulin resistance and hypercholesterolemia induced by feeding high fructose diet in rats. Yoshinaga et al. [91] found that administrating of 1 g of wakame (*Undaria Pinnatifida*) per 100 g diet significantly decreased serum total cholesterol levels and lead to a reduced accumulation of body fat in rats. However, in both studies identification of the components from the algae responsible for the results was not determined.

### 2.3. Fish Oil 

**Figure 3:**
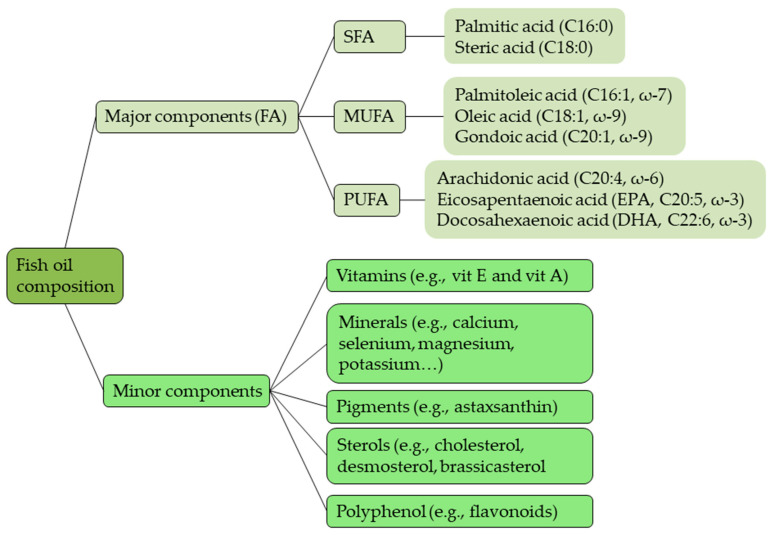
Composition of fish oils.

The consumption of marine fish and seafood has been associated with many health benefits, mostly from the uptake of fish oil. Fish oil owe their special properties main to the principally ω-3 FA, which include long-chain (LC) ω-3 PUFA, mainly eicosapentaenoic acid (EPA) and docosahexaenoic acid (DHA), which have beneficial effects on human health (Figure 3) [92,93,94]. 

Fish oil has been used in the food, biomedical, and pharmaceutical sectors. Several studies have reported that fish oil can be used to treat various disorders and prevent the progression of several chronic diseases (Table 1) [95,96]. Fish oil can be used for human consumption either by directly eating fish (and algae) or by consuming different formulations, such as tablets or capsules [92,97]. Previous studies have shown that fish oil enriched with EPA and DHA can be applied in the form of capsules to prevent CVD, by reducing the risk of high TG, hypertension, dyslipidemia, heart disorders, while decreasing blood levels of low density cholesterol [95,98]. The regular use of fish oil capsules can help prevent cancers, especially for patients with progressive cancers [93,96]. Fish oil enriched with ω-3 FA has been recommended to optimize the functions of the human brain, kidney, liver and heart, and hence, to decrease the progression of cardiovascular, hypertension, cancer, neurodegenerative, auto-immune and renal diseases [96,99]. Also, fish oil enriched with LC ω-3 PUFA can reduce the amounts of C-reactive protein (CRP) and pro-inflammatory cytokines, and thus, reduce the risk of inflammatory diseases, such as rheumatoid arthritis [95,100,101]. De Souza et al. [102] have reported that the FA of fish oil capsules can be used for the treatment of other disorders, such as type-2 diabetes mellitus (T2DM) and obesity, and also to decrease the atherogenic factors, principally the atherogenic index of plasma (AIP) in patients with T2DM who suffer from obesity. Earlier studies have reported that fish oil can be used to lower the risk of Alzheimer’s disease [95]. 

### 2.4. EAA in Protein Supplement Systems 

**Figure 4:**
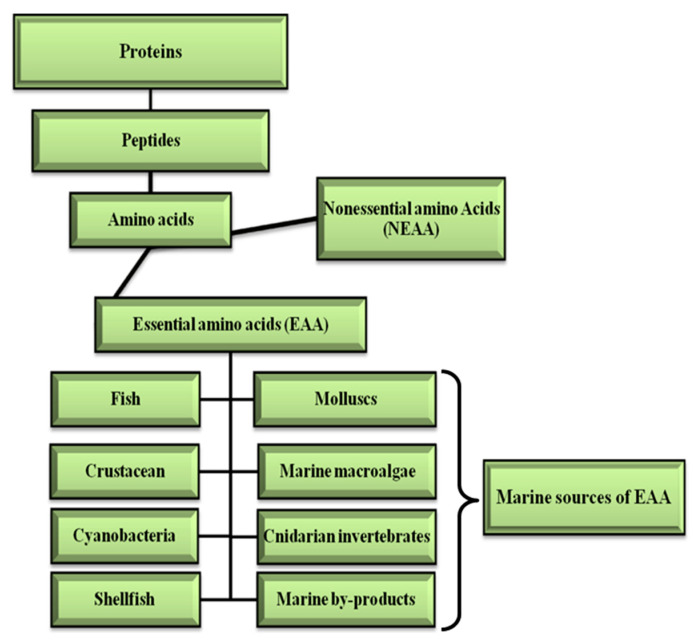
Schematic representation of different marine sources of essential amino acids (EAA), as functional ingredients from proteins and peptides.

Proteins have several important functions in living system such as protecting the immune system, the storage and transit of other molecules, and also as catalysts [103]. Marine organisms have bioactive proteins and peptides which also provide EAA. Crustaceans, fish and molluscs are important sources of EAA, such as arginine, leucine, isoleucine, gamma-aminobutyric acid (GABA), glycine, glutamic acid, methionine, and phenylalanine (Figure 4) [104,105,106]. 

The EAA also have antihypertensive, antibacterial, antioxidant, hypocholesterolemic, hypoglycemic, anti-coagulant and immunomodulatory activities (Table 2). 

**Table 1:** Overview of some research studies regarding fish oils and their potential health benefits.

Fish Oils	Functional Substances		Beneficial Effects in Human Health and Pharmaceutical Properties	References
Krill oil	Fatty acids	- PUFA: ω-3 EPA and DHA (~40%)	- Anti-cardiovascular - Anti-obesity - Anti-inflammatory	[107]
	Vitamins	- Vitamin A - Vitamin E	- Immunomodulatory - Antioxidant	[108]
	Pigments	- Carotenoids: Astaxanthin	- Antioxidant	[108]
Tuna oil	Fatty acids	- PUFA: ω-3 EPA (7.81%) and DHA (24.56%) - MUFA: ω-9 oleic acid	- Anti-cardiovascular (prevention and treatment of hypertension and arteriosclerosis) - Anti-inflammatory properties. - Prevention of mitochondrial dysfunction. - Insulin resistance in skeletal muscle and neuronal cells - Reduction of the accumulation of visceral fat - Anti-obesity	[108,109,110,111]
	Vitamins	- Vitamin A - Vitamin D	- Immunomodulatory - Anti-oesteomalacia - Anti-rickets	[110,112]
Mackerel oil	Fatty acids	- PUFA: ω-3 EPA and DHA - ω-6 arachidonic acid	- Anti-inflammatory - Pro-inflammatory - Photoprotective properties. - Prevention of erythema - Prevention of cardiovascular diseases	[110,112,113,114]
	Vitamins	- Vitamin A - Vitamin D	- Immunomodulatory - Anti-oesteomalacia - Anti-rickets	[110,112]
Salmon oil	Fatty acids	- PUFA: ω-3 high levels of EPA and DHA - MUFA: ω-9 oleic acid	- The decrease of cholesterol and triglyceride in plasma, as well as the amounts both of LDL and VLDL, in normolipidemic subjects. - Reduction of risk cardiovascular diseases. - Improve the normal brain function and development - Anti-hypertensive - Prevention of adrenoleukodystrophy diseases	[115,116,117,118,119]
	Vitamins	- Vitamin A - Vitamin D	- Immunomodulatory - Anti-oesteomalacia - Antirickets	[108,110]
	Pigments	- Carotenoids: astaxanthin	- Antioxidant - Anti-diabetic - Anti-cancer - Anti-inflammatory - Anti-tumor	[120]
Sardine oil	Fatty acids	- PUFA: ω-3 high amounts of EPA and DHA - MUFA: ω-9 oleic acid	- Anti-cardiovascular - Regulation of blood cholesterol amounts - Promotion of heart health - Protection of the arteries walls. - Promotion of cardiovascular health - Anti-inflammatory - Anti-cancer - Photoprotective effect - Prevention of erythema - Anti-obesity	[121,122,123]
	Vitamins	- Vitamin D	- Anti-oesteomalacia - Anti-rickets	[110]
Herring oil	Fatty acids	- PUFA: ω-3 higher levels of EPA and DHA	- Anti-cardiovascular properties. - Anti-cancer - Treatment of cutaneous infection, melanogenesis and dermatitis. - Anti-inflammatory - Inhibition of bacterial infection (protection against *S. aureus*) - Antioxidant properties	[112,119,124,125]
Menhaden oil	Fatty acids	- PUFA: ω-3 high levels of EPA and DHA	- Immunomodulatory - Prevention of coronary heart diseases - Prevention of cardiovascular diseases - Prevention of lymphoproliferative diseases - Treatment of diabetic conditions	[112,126,127,128]
	Vitamins	- Vitamin A - Vitamin D	- Immunomodulatory - Regulation of phosphorus and Ca - Homeostasis	[108,129]

**Table 2:** EAA obtained from marine sources and their potential health benefits.

Essential Amino Acid (EAA)	Examples of Marine Sources	Beneficial Effects in Human Health and Pharmaceutical Properties	References
Arginine (Arg)	- Fish: *Caranx ignobilis, Neolissochilus hexagonolepis, Labeo rohita, Tor putitora, Clarias batrachus, Anabas testudineus, Oncorhynchus mykiss* - Molluscs: Oysters, Box jellyfish (*Cubozoa* sp.) - Cyanobacteria	- Antihypertensive, antioxidant and immunomodulatory - Enhance of growth, cell division and neurotransmission - Role in the mechanism of hormone secretion - Treatment of disorders such as anxiety, preeclampsia, and sepsis	[130,131,132,133]
Histidine (His)	- Fish: skipjack tuna (*Katsuwonus pelamis*), *Nemipterus japonicus, Labeo rohita, Stolephorus commersonii, Catla catla, C. batrachu, Cirrhinus mrigala, Anabas testudineus, Amblypharyngodon mola, Rastrelliger kanagurta, Puntius sophore* - Crustaceans: *Charybdis natator* - Marine macroalgae: *Ulva* sp., *Gracilaria* sp.	- Tissue repair, role in the growth, protection of the myelin sheaths. - Eliminates dangerous metals from the body. - Antioxidant and anti-inflammatory properties - Prevention of risk factors of prediabetes. - Treatment of neurological diseases, rheumatoid arthritis, ulcers, malignancies, anemia, atopic dermatitis, ocular system - Metabolic regulation and modulation of intestinal cell.	[131,134,135,136]
Isoleucine (Iso)	- Fish: *N. japonicus, L. rohita, Catla catla, C. mrigala, A. testudineus, T. putitora, O. mykiss, R. kanagurta, S. commersonii, Thunnus albacares, Stolephorus waitei*	- Important role in the muscle formation, normal growth and development, synthesis of cellular proteins and production of β-defensins - Regulation of diabetes conditions, diver’s metabolisms. - Stimulation of mitochondrial biogenesis - Antibacterial an anticancer properties.	[131,137]
Leucine (Leu)	- Fish: *Sardina* spp., *K. pelamis, L. rohita, S. waitei, S. commersonii, R. kanagurta, N. japonicus, T. albacares, C. catla, L. rohita, Heteropneustes fossilis, C. batrachus, Siberian sturgeon, Silurus glanis* - Molluscs: *Anadara broughtonii, Mactra chinensis*	- Anticancer, anti-obesity properties - Regulation of the function and activity of lymphocytes. - Modulation of gene expression. - Improvement of the growth and development of skeletal muscles and small intestine, stimulation of protein synthesis. - Treatment of stress conditions, such as trauma, burn, and sepsis.	[130,131,137,138]
Lysine (Lys)	- Fish: *K. pelamis, T. albacores, S. commersonii*, C. batrachus, *Anabas testudineus, Cirrhinus mrigala, S. sturgeon, T. putitora, Bighead carp, S. glanis* - Molluscs: *A. broughtonii, M. chinensis*	- Important role in the development and growth. - Immunomodulatory. - Antitumor, antimicrobial, antioxidant properties	[130,131,134,138,139,140]
Methionine (Met)	- Fish: *K. pelamis, T. putitora, Stolephorus waitei, Rastrelliger kanagurta*	- Treatment of liver diseases, depression, asthma, allergies, alcoholism, copper poisoning, Parkinson, schizophrenia - Enhancing wound healing.	[130,131]
Phenylalanine (Phe)	- Fish: *K. pelamis, C. catla, C. mrigala, L. rohita*, Grass carp (*Ctenopharyngodon idella*), *S. sturgeon, S. glanis* - Crustaceans: Shrimps	- Regulation of diabetic conditions - Development of muscles. - Antioxidant activity.	[130,131,141]
Tryptophan (Trp)	Fish: *Sardina* spp., *K. pelamis*, *Thunnus* sp., *T. putitora*	- Regulation of neurological system. - Important role in the function of neurotransmitters, such as nor-dopamine and dopamine. - Treatment of insomnia, depression, pain, seasonal affective, hyperactivity, dysphoric	[130,137]
Valine (Val)	- Fish: *Thunnus* sp., *Pseudocaranx* sp, *K. pelamis*, C. idella - Crustaceans: Shrimps - Molluscs: Oysters, Cuttlefish (*Sepia officinalis*)	- Anticancer, anti-inflammatory properties - Immunomodulatory.	[130,137]
Proline (Pro)	- Fish: *Thunnus* sp., *K. pelamis*, *Salmoninae*, *Raja* sp., *C. idella* - Molluscs: *S. officinalis*	- Modulation of gene expression. - Improving the growth of skeletal muscle and also the small intestine. - The decrease of excessive body fat.	[130,137]
Glycine (Gly)	- Fish: *Cirrhinus mrigala, Labeo rohita, C. catla, Raja* sp., - Molluscs: *Anadara broughtonii, Mactra chinensis*	- Regulation and function of neurological system, metabolism mechanisms and gene expression. - Antioxidant, anti-cancer, anti-inflammatory and anti-obesity properties. - Important role in the protein synthesis. - Enhancing the immune system. - Treatment of metabolic diseases and diabetes conditions. - Prevention of cardiovascular disorders	[130,137,142,143]

EAA supplements of “cysteine, leucine, histidine, methionine, proline, hydroxyproline, tyrosine, threonine, trans-4-hydroxy-proline, and valine” showed an antioxidant activity due to radical scavenging activity and lipid peroxidation inhibition [104,144], and help with human homeostasis, principally due to their function in the regulation of various cellular mechanisms and as precursors of other molecules (e.g., nitrogenous bases and hormones) and also as protein building blocks [144].

Some EAA derived from the two mollusks *Rapana venosa* and *Mytilus galloprovincialis* (L.) showed good anti-inflammatory activity [145]. 

GABA as an EAA produced from marine organisms, such as marine cyanobacteria, is a neurotransmitter inhibitor and can decrease hypertension by decreasing blood pressure. GABA can also stimulate the immune systems to help treat autonomic diseases and depression, and regulate diabetes by its anti-hyperglycemic effect [104,146]. 

On the other hand, the natural bioactive EAA taurine, which can be derived from crustaceans and mollusks is a “β-amino-sulphonic acid”. Taurine has shown several physiological and biological properties in humans, such as the stabilization of cell membranes, helping the development of the retina and central nervous system, and immunomodulatory effects [104].

### 2.5. Minerals in Seafood for Human Diet 

Seafood can be a rich source of essential minerals in the human diet. Although the flesh of fish and other seafood can be a good source of Ca, phosphorus, magnesium, zinc, iron, selenium and iodine [147,148], even higher levels of minerals can be obtained from the seafood industry’s by-products.

Ca is the main mineral obtained from seafood by-products, mainly fish bones and shells [149,150]. Ca in shells is usually present in the form of calcium carbonate while the Ca from bones is mostly present in the form of hydroxyapatite or tricalcium phosphate [151,152]. Ca from shells is usually obtained through the process of calcination, during which the shell, which is mostly argonite, is heated to obtain specific structural changes. During heating >500 °C the argonite structure is reorganized into the triagonal-rhombohedral structure of calcite and calcium oxide (CaO) if the temperature is increased to >600–800 °C [152]. The calcinations of fish bones using temperature in the range of 600–1200 °C results in calcium phosphates in the form of hydroxyapatite and tricalcium phosphate. The higher the temperature of calcination the higher the rate of transformation of hydroxyapatite to tricalcium phosphate [153]. Those compounds have a number of health benefits and can be used in tissue engineering scaffolds, implants, dietary supplements or food additives. Both hydroxyapatite and tricalcium phosphate have been used in bioceramics to produce scaffolds for tissue engineering and bone regeneration as well as substrates for coatings of metallic implants [154,155]. Hydroxyapatite can be also a valuable source of dietary supplements for humans, with higher efficiency and tolerability then commonly used calcium carbonate [156] and no observed acute or chronic toxicity in regular and nanoparticle size [157,158].

Although fish bones consist mostly of Ca and P, which constitute >95% of fish bone minerals, various other microelements are also obtained during the preparation process, resulting in a product with potential food supplement applications [159]. Bubel et al. [160] developed a simple method for Ca preparations from cod and salmon backbone containing 24.9–27.8% Ca and 12.5–13.4% P but also relatively high levels of Mg (4.6–6.6 g/kg) and microelements: 3.9–6.2 mg/kg Cu, 11–24 mg/kg Fe, 28–53 mg/kg Mn and 50–57 mg/kg Zn. Even higher levels of Ca (38.2%) and P (23.3%) have been found in tuna bone powder. Aside from those two elements the bone powder also contained relatively high levels of Fe (62 mg/kg) and Mg (4700 mg/kg) [161]. As reported by Flammini et al. [162], boiling hake bones further increased the Ca and P content of the bone powder, while significantly decreasing the Na and K content. Such powder showed good cell bioavailability and resulted in significant improvement of rat bone mineralization, comparable to the improvements observed for commercial supplements. 

The bioavailability of Ca from fish bone is correlated with its size and solubility [163,164]. Moreover, reducing the particle size of bone powders to nanoscale can improve the bioavailability even further [165,166], while the tests with rats showed no observed adverse effect level of nano calcium carbonate [167].

Aside from fish bones and seafood shells, no other seafood by-product is being used as a potential source of minerals. On the other hand, there are a number of fish by-products, such as fish viscera, which might provide a valuable amount of minerals if properly extracted, since the ash content of fish viscera ranges from 7–11% of dry weight [168,169], with high content of Ca, K and Mg [170]. However, there is little data regarding the exact mineral composition of the fish viscera, providing a field for future research.

### 2.6. Marine-Based Vitamin Sources

Seafood can be a valuable source of all vitamins necessary for the human diet, especially vitamin A, D, E and B_12_ [171,172] and, in case of some algae even vitamin C [173]. Seafood as a source of vitamins have been thoroughly reviewed [110,174,175], therefore this section will focus on recent results. The seafood lipid fraction is an important source of not only ω-3 FA but also high levels of fat-soluble vitamins [176]. A summary of recent results on seafood-based fat-soluble vitamin sources is shown in Table 3.

**Table 3:** Recent results on seafood-based fat-soluble vitamin sources.

Vitamin	Source	Key Findings	Reference
E	Crude oil from farmed tuna liver Crude oil from farmed tuna gill and gut Crude oil from sardine heads, gut and fins Crude oil from whole sardine Crude oil from farmed seabass and seabream heads and gut	Significantly lower α-tocopherol in all crude oils then in cod liver oil. Oil from tuna by-products had similar α-tocopherol as tuna liver oil Crude oil from sardine by-products had significantly higher α-tocopherol then crude oil from whole sardines No correlation found between higher α-tocopherol content and crude oil stability	[177]
	Cod liver oil	Refining of crude oil resulted in 31–45% decrease in α-tocopherol	[94]
	Oil from rainbow trout heads, bones and tails Oil from rainbow trout intestines	The oil extraction temperature did not affect α-tocopherol of different oils The α-tocopherol level in oils ranged from ~90–160 µg/g of oil	
	Fresh *Caulerpa* sp. leaves	Vitamin E content of 2.2 mg/kg	[178]
	Rainbow trout flesh	Out of 5 extraction methods of α-tocopherol, solid-liquid extraction with n-hexane showed the best performance	[179]
K	Meat of Atlantic salmon fed a diet with high vitamin D_3_ and K_1_	Improvement in several bone formation and resorption markers after consuming salmon fed with high vitamin D and K. The results were obtained despite using vitamin K_1_ for supplementation	[180]
D	Anchovy filleting wastes	Oil extracted using d-limonene as biosolvent contained 81 µg of vitamin D_3_/kg of oil	[181]
	Wakame and combu leaves	Vitamin D <0.05 µg/100 g in both fresh and dried leaves	[182]
A	Pangasius catfish filleting wastes	Fish oil obtained as part of a zero-waste procedure, contained 334 µg of retinol/kg of oil	[183]
	Fresh *Caulerpa sp.* leaves	High vitamin A reaching 4810 mg/kg	[178]
	Dried *Ulva lactuca*	Vitamin A below detection limit	[184]

Despite the multiple functional roles of vitamin D in humans, its deficiency has been reported in many different populations worldwide [185,186,187,188]. Seafood, mainly oily fish species or fish liver from non-fatty fish, are the main source of vitamin D in the human diet, since it is the only food product, other than mushrooms and egg yolk, that have this vitamin at relatively high levels [186]. Special care should be taken when selecting a type of seafood as a source for vitamin D, since many processed seafood products, such as fish fingers (or sticks), do not contain high levels [189]. Some edible seaweeds have high levels of vitamin D_2_, while others, like combu and wakame, have levels below the detection limit (0.05 µg/g) [182]. Fortifying foods with vitamin D has shown positive outcomes in both children and adults [190,191,192]. However, Jahn et al. [193] indicated that not all products fortified with vitamin D have met with a positive consumer response. To successfully use food fortification as a tool to fight vitamin D deficiency, the following issues have to be addressed: consumers have to have a positive attitude towards the food product, consumers have to see a personal benefit from the fortified food, have cultural appropriateness and have an awareness of the prevalence of vitamin D deficiency in society. 

An economically viable option can be to obtain this vitamin in fish oil from seafood by-products. To improve the quality of extracted fish oil, recent studies have used biosolvents such as d-limonene, instead of traditional organic solvents. D-Limonene is nontoxic and can be fully recovered by hydrodistillation below 100 °C [194]. It has been used to obtain high quality fish oil from anchovy filleting waste with a vitamin D_3_ content of 81 µg/kg [181,195]. 

Seafood, especially seaweeds, can be a valuable source of vitamin A [55,196]. For example, *Caulrepa sp.* have high levels of vitamin A (4.8 g/kg) [178]. On the other hand, not all seaweeds are a rich source of vitamin A, as shown by Rasyid [184], who reported that vitamin A in dried *Ulva lactuca* was below the detection limit. Vitamin A can also be obtained from fish and its by-products. Nam et al. [183] used an enzymatic hydrolysis of Pangasius catfish filleting by-products which included head, trimmings, viscera, scales, liver, roe and skin, during a zero-waste procedure to obtain protein hydrolysate, hydroxyapatite and, after additional purification, fish oil with a vitamin A content of 334 µg/kg of oil. The vitamin A content, however, was relatively low, when compared to the retinol content in an unspecified fish wastes oil reported by Tyśkiewicz et al. [197] (0.70 g/kg).

Vitamin E is commonly associated with four tocopherols and four tocotrienols (α, β, γ and δ). This may, however, be inaccurate, since only α-tocopherol fulfills the definition of a “vitamin”, while the other tocopherols and tocotrienols do not prevent ataxia, a vitamin E deficiency symptom [198]. Fortunately, α-tocopherol is the main tocopherol found in seafood products [176].

α-Tocopherol is often obtained from seafood during crude oil extraction. Having strong antioxidant properties, α-tocopherol is often responsible for better oil stability during storage [199,200]. Although in general low temperature oil extraction yields better stability of fish oil, Honold et al. [201] observed, that different oil extraction temperatures (70 vs. 90 °C) did not affect the α-tocopherol content of the oil extracted from rainbow trout by-products (head, bone, tail and intestine), with the content ranging from ~90 to 160 µg/g of oil. Crude oils with high levels of α-tocopherol can be obtained from various fish by-products. For example, Šimat et al. [177] used tuna gill and gut to obtain crude oils with a high yield of 26.1%, which was an α-tocopherol content of 76 µg/g. On the other hand, extraction of crude oil from sardine head, gut and fin resulted in a yield of only 9.2% which was 36 µg/g of α-tocopherol. Therefore, the choice of fish species and type of by-product are important factors to recover high levels of α-tocopherol. Processing of crude oil also negatively affected the α-tocopherol content, as reported by Šimat et al. [94] who observed a 31–45% reduction in α-tocopherol content after refining of crude oil from fish by-products. Araújo et al. [179] compared 5 different methods of vitamin E extraction from rainbow trout flesh: Soxhlet extraction, Folch extraction, solid-liquid extraction with n-hexane and with methanol-BHT, and saponification with KOH with magnetic agitation and n-hexane extraction. They determined that the solid-liquid extraction with n-hexane was the most suitable method after further optimization.

The typical recommended daily intake (RDI) for vitamin K varies depending on the source. The typical recommendations are in the range of 55–75 µg/day (~1 µg/kg of body weight/day), however, some sources recommend a daily intake of ≤600 µg/day [202]. Usually fish are not a good source of vitamin K, with its content measured as a sum of K_1_ and K_2_ usually within the range of 1.8–11.3 µg/kg. However, there are seafood sources with much higher vitamin K content such as eel (644 µg/kg) or dried seaweeds (1750–12,900 µg/kg) [203,204]. Both microalgae and macroalgae have been suggested as a good source for sustainable production of vitamin K_1_ [205,206]. On the other hand, fish usually contains vitamin K in the form of menaquinone (K_2_) while algae are a source of phylloquinone (K_1_) and those two form of vitamin K often show different functions and health promoting properties, therefore algae should not substitute for fish or other animal-based food products as a source of vitamin K, but rather complement it [207].

Graff, et al. [180] have investigated the use of Atlantic salmon fed with a high vitamin D_3_ and K_1_ diet on several bone formation markers of human subjects with additional Ca supplementation. They found that the consumption of such treated salmon improved more significantly the bone formation markers then in patients consuming salmon fed only high vitamin D_3_. Surprisingly, these results were observed even though the vitamin K was in the form of phylloquinone, meanwhile the positive effect on bone quality is usually associated with menaquinone [202]. 

Seafood can also be a valuable source of water-soluble vitamins. One of the most important water-soluble vitamins found in seafood is vitamin B_12_, which can be found mainly in sources of animal origin or algae, with the latter being a potential source of vitamin B_12_ for food fortification and supplementation [208]. Seafood has been found to be the main source for vitamin B_12_ in the diet of adult Koreans and Canadians [209,210]. The dark meat of fish muscle usually contains a few times higher levels of vitamin B_12_ then the light meat. Moreover, fish by-products, such as viscera, are also a potential resource for vitamin B_12_ extraction, since they can contain significantly higher levels of this vitamin then the muscle [211]. 

Seafood can be a valuable source of vitamins, which are often hard to supply from other food sources. Those include vitamin D or B_12_. As in case of minerals, the seafood by-products seem to be a potential source of vitamins.

### 2.7. Dopamine in Seafood as Drug and Supplement

**Figure 5:**
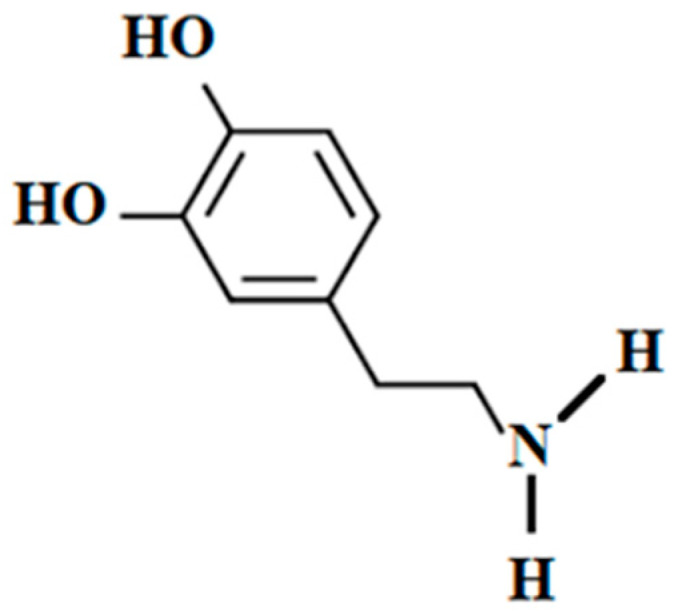
Chemical structure of dopamine.

Dopamine (3,4-dihydroxyphenethylamine, DA: Figure 5) is a neuroendocrine transmitter and could also be used as a drug. It has an important role in brain functions [212]. Motivation, dreaming, sleeping mood, punishment, learning, attention, and memory are some of the areas impacted [213,214]. Also, the drugs obtained from DA or its metabolites are being utilized to control clinical disorders (bipolar disorder, Parkinson disease, and different types of addiction, etc.) [215,216,217,218,219]. Besides these functions of DA on brain functions, Pacifici [220] reported that DA could be used to increase blood pressure and urine output as well as for pediatric treatments [221]. The sourcing of DA is potentially important to the drug industry. 

Cephalopods like squid, cuttlefish, and octopus have black inks, which are composed of melanin, some enzymes (tyrosinase), some amino acids, and DA. Cephalopods could be evaluated as a source of DA although there are some inkless octopus species [222,223,224,225]. Squid ink has anticancer, antioxidant, anti-retroviral, and antimicrobial activities as described by Jismi et al. [226]. Palumbo et al. [227] showed that squid ink contains a significant amount of DA. Also, Lucero et al. [228] implied that the concentration of L-DA and DA in squid ink were 1.15 and 0.19 mM DA, respectively. Cuttlefish (*Sepia officinalis*) ink is also a good source of DA in various forms. According to HPLC analysis results based on crude ink obtained from the whole cuttlefish, the concentrations of dopa and DA were found to be 2.2 ± 0.8 and 0.06 ± 0.02 nmol/mg of protein, respectively [229]. DA is obtained from L-tyrosine protein-rich foods. Therefore, the concentration of DA in the protein fraction should be measured. In addition to some cephalopod inks, Naila et al. [230] reported that DA could be found in fish, meat, and their products. Thus, these protein-rich foods could be evaluated as options for a person who wants or needs to increase DA levels, i.e., the brain needs tyrosine in protein-rich foods like fish to make DA. 

For acute correction of hemodynamics in shock states, a DA hydrochloride and 5% dextrose injection could be used. Use of 800, 1600, or 3200 mcg/mL doses of DA by infusion depended on the patient’s body weight from 10 to 100 kg. Traumatic brain injury (TBI) is one of the leading causes of death. TBI has been associated with fluctuations in DA levels [231]. 

Eating a balanced diet is important for maintaining the DA level in blood. However, if DA is urgently needed, it could be injected. Probiotics, some minerals, vitamins, and fish oil supplements might also be used to help boost DA but have been limited because non-prescription approaches have not been promoted. For example, consumption of squid, cuttlefish or octopus ink soups might be appropriate. Pills with DA produced from squid, cuttlefish, and octopus inks might be beneficial. 

### 2.8. Bioactive Peptides from Marine Sources

Marine organisms such as sponges, tunicates, bryozoans, mollusks, bacteria, microalgae, macroalgae, cyanobacteria, fish, and crustaceans have bioactive peptides with properties such as antimicrobial, cardioprotective (anticoagulant, antihypertensive, antiatherosclerotic), antioxidant, radioprotective, antiparasitic, anti-inflammatory, and anti-cancerous activities [58].

The protein component of fish and other marine organisms and their by-products have ingredients with potentially important roles as functional and medicinal foods that may prevent and/or treat many chronic diseases. Because of the presence of all EAA in significant amounts, the marine macroalgae are a potential source of high-quality proteins with better nutritional properties than terrestrial plants [232]. A number of proteins, peptides, and amino acids from marine organisms can reduce inflammation thus improving the immune system against factors such as bacterial/viral infection, and injury [135,233]. Marine proteins are being studied for anticancer therapy. Evidence of new cytotoxic proteins, such as chondroitin identified from a common marine sponge *Chondrosia reniformis* [234] have been reported.

Bioactive peptides are inactive in their parent protein but can be released when large pre-propeptides are broken down to specific protein fragments and modified to have numerous beneficial effects to improve the physiological functions of the body [5]. The best sources of structurally diverse bioactive peptides with functions such as ACE inhibitory and anti-hypertensive, antioxidative, anticoagulant, and antimicrobial effects are marine organisms. Marine bioactive peptides may be produced by solvent extraction, enzymatic hydrolysis, or microbial fermentation of proteins, resulting in fragments that usually contain 3–20 amino acid residues. Their amino acid sequence determines the biological activity. The molecular size and structural characteristics of peptide mixtures in protein hydrolysates contribute to their bioactivity, and low MW fractions (1 to 5 kDa) in general contain more potent antioxidative peptides [235]. Peptide fractions can be separated using column chromatography to obtain pure peptides [236]. For example, those with a tripeptide sequence at the C-terminal end of peptides with antihypertensive activity contain hydrophobic amino acid. They have been shown to be ACE-inhibitory peptides [130]. ACE causes blood vessels to constrict increasing blood pressure. Commercial ACE inhibitors (benazepril, captopril, enalapril, perindopril, trandolapril, quinapril, lisinopril and moexipril) produce side effects such as coughs, increased blood potassium levels, low blood pressure, skin rashes, headaches, fatigue, fetal and taste disorders [130]. Natural components with ACE inhibitory capacity have become a focus of hypertension treatment studies. The peptide sequence of ACE-inhibitory peptides from marine organisms with strong ACE inhibitor capacity have been studied (Table 4) and their activity described [5,130,237,238,239]. 

A cause of many health disorders, such as diabetes, neurodegenerative and inflammatory diseases, and cancer reflect uncontrolled production of ROS that attack macromolecules such as membrane lipids, proteins, and DNA resulting in cellular or tissue level injuries [240]. These health conditions and/or their symptoms can be prevented through the antioxidant effects of functional and medicinal foods [232]. Lipid oxidation is also a major cause of food quality deterioration that leads to rancidity and formation of undesirable lipid peroxidation products, such as malondialdehyde. Oxidation decreases both the sensory and nutritive quality of foods. Lipid oxidation has long been recognized as a major problem during the handling, processing, and distribution of PUFA-rich foods. Natural antioxidants may serve as an alternative to the synthetic antioxidants (propyl gallate, butylated hydroxytoluene, butylated hydroxyanisole, and tert-butylhydroquinone) used to control lipid oxidation but restricted because of their induction of DNA damage and potential toxicity [1].

Antioxidant peptides have been isolated from a range of marine organisms (Table 4), from the smallest marine rotifer (*Brachionus rotundiformis*) [241], oysters (*Crassostrea gigas*) [234], different marine vertebrates and invertebrates [236], and marine by-products [235,242]. The antioxidant properties of peptides are commonly determined based on different in vitro assays, e.g., scavenging of free radicals (2,2-diphenyl-1-picrylhydrazyl, hydroxyl, superoxide), reducing ferric iron to ferrous, binding of metals (metal chelation), and inhibiting lipid oxidation. However evidence of the in vivo antioxidant capacity as well as cellular studies of peptides are a necessary step before human clinical trials [236]. The biological potential of marine antioxidant peptides with human clinical trials is limited [243].

**Table 4:** An overview of recent studies on the biological activity of marine-originated peptides.

Marine Source	Biological Activity	Amino Acid Sequence	Reference
Cuttlefish (*Sepia officinalis*)	ACE inhibitory	Val-Glu-Leu-Tyr-Pro	[244]
Flounder fish (*Paralichthys olivaceus*)	ACE inhibitory	Met-Glu-Val-Phe-Val-Pro	[245]
Lizard fish	ACE inhibitory	Gly-Met-Lys-Cys-Ala-Phe	[246]
Pacific cod (*Gadus macrocephalus*)	ACE inhibitory	Gly-Ala-Ser-Ser-Gly-Met-Pro-Gly and Leu-Ala-Tyr-Ala	[247]
Shrimp paste	ACE inhibitory	Ser-Val and Ile-Phe	[248]
Jellyfish (*Rhopilemae sculentum*)	ACE inhibitory	Gln-Pro-Gly-Pro-Thr and Gly-Asp-Ile-Gly-Tyr	[249]
Marine snail (*Cenchritis muricatus*)	Antifungal activity	Ser-Arg-Ser-Glu-Leu-Ile-Val-His-Gln-Arg	[250]
*Spirulina maxima*	Anti-atherosclerotic activity	Leu-Asp-Ala-Val-Asn-Arg and Met-Met-Leu-Asp-Phe	[251]
*Pyropia yezoensis*	Anti-inflammatory activity	Lys-Ala-Gln-Ala-Asp	[252]
Skate (*Okamejei kenojei*)	ACE inhibitory	Leu-Gly-Pro-Leu-Gly-His-Gln and Met-Val-Gly-Ser-Ala-Pro-Gly-Val-Leu	[253]
Dulse (*Palmaria palmata*)	Renin inhibitory, Antihypertensive effect	Ile-Arg-Leu-Ile-Ile-Val-Leu-Met-Pro-Ile-Leu-Met-Ala	[254]
Half-fin anchovy (*Setipinna taty*)	Pro-apoptotic on PC-3 cells	Tyr-Ala-Leu-Arg-Ala-His	[255]
Greater pipefish (*Syngnathus acus*)	Pro-apoptotic on A549 and CCRF-CEM cells	Lys-Arg-Asp-Leu-Gly-Phe-Val-Asp-Glu-Ile-Ser-Ala-His-Tyr	[256]
Japanese flounder (*Palatichtys olivaceus*)	Antioxidative activity	Gly-Gly-Phe-Asp-Met-Gly	[257]
Nori (*Porphyra yezoensis*)	Anticoagulant activity	NH2-Asn-Met-Glu-Lys-Gly-Ser-Ser-Ser-Val-Val-Ser-Ser-Arg-Met-Lys-Gln-COOH	[258]
*Porphyra haitanesis*	Anti-proliferation activity	Val-Pro-Gly-Thr-Pro-Lys-Asn-Leu-Asp-Ser-Pro-Arg and Met-Pro-Ala-Pro-Ser-Cys-Ala-Leu-Pro-Arg-Ser-Val-Val-Pro-Pro-Arg	[259]
Dulse (*Palmaria palmata*)	Antioxidant activity	Ser-Asp-Ile-Thr-Arg-Pro-Gly-Gly-Asn-Met	[260]
Laver (*Porphyra spp*)	α-Amylase inhibitory activity	Gly-Gly-Ser-Lys and Glu-Leu-Ser	[261]
Atlantic salmon (*Salmo salar*)	Anti-allergic activity	Thr-Pro-Glu-Val-His-Ile-Ala-Val-Asp-Lys-Phe	[262]
Fermented anchovies (*Ilisha melastoma*) sauce (Budu)	Antioxidant activity	Lue-Asp-Asp-ProVal-Phe-Ile-His	[263]
Blood cockle (*Tegillar cagranosa*)	Antioxidant activity	Met-Asp-Leu-Phe-Thr-Glu and Trp-Pro-Pro-Asp	[264]
Mackerel (*Scomber japonicus*)	Antioxidant activity	ALSTWTLQLGSTSFSASPM	[243]
Oyster (*Crassostrea gigas*)	Antioxidant activity	Leu-Lys-Gln-Glu-Leu-Glu-Asp-Leu-Leu-Glu-Lys-Gln-Glu	[234]
Marine crab (*Charybdis natator*)	Anti-inflammatory effect	G-L-G-A-A-V-L	[135]
Red scorpionfish (*Scorpaena notata*)	ACE inhibitory and antioxidant activity	Gln-Gln- Pro-His-Ser-Arg-Ser-Lys-Gly-Phe-Pro-Gly-Pro, Gly-Gln-Lys-Ser-Val-Pro-Glu-Val- Arg and Val-Glu-Gly-Lys-Ser-Pro-Asn-Val	[265]
Pearl oyster (*Pinctada fucata martensii*)	ACE inhibitory	His-Leu-His-Thr, and Gly-Trp-Ala	[266]
Spotless smoothhound (*Mustelus griseus*)	Antioxidant activity	Gly-Ala-Glu-Arg-Pro, Gly-Glu-Arg-Glu-Ala-Asn-Val-Met and Ala-Glu-Val-Gly	[267]
*Tetradesmus obliquus* microalgae	Antioxidant and ACE-inhibitory activity	Trp-Pro-Arg-Gly-Tyr-Phe-Leu, Gly-Pro-Asp-Arg-Pro-Lys-Phe-Leu-Gly-Pro-Phe, Trp-Tyr-Gly-Pro-Asp-Arg-Pro-Lys-Phe-Leu and Ser-Asp-Trp-Asp-Arg-Phe	[268]
Nile tilapia (*Oreochromis niloticus*)	Antimicrobial activity	Phe-Ile-His-His-Ile-Ile-Gly-Gly-Leu-Phe-Ser-Ala-Gly-Lys-Ala-Ile-His-Arg-Leu-Ile-Arg-Arg-Arg-Arg-Arg	[269]
Sea cucumber (*Stichopus japonicus*)	ACE inhibitory	Asn-Ala-Pro-His-Met-Arg	[270]
Sponge (*Xestospongia testudinaria*)	Cytotoxic to cancerous HeLa cells	Lys-Glu-Asn-Pro-Val-Leu-Ser-Leu-Val-Asn-Gly-Met-Phe	[271]

#### Gelatin from Marine Sources

Gelatin is not a naturally occurring protein. It is, however, one of the most versatile biopolymers, an animal-based protein obtained by thermal denaturation with partial acid/alkaline hydrolysis or enzymatic hydrolysis of collagen. The marine-originated gelatin is primarily composed of crude protein, moisture, and ash, with some carbohydrates. Its properties are different from those from animals sources which has found some suitable applications in the food and pharmaceutical industries, but also for medicinal purposes [272,273]. In 2018 the global gelatin market was estimated at 4.5 billion USD [274], with an increased demand for gelatin from sources other than bovine and porcine. The raw materials commonly used for gelatin production are the bones, skins, and connective tissue of terrestrial animals such as cattle and pigs. The need for new sources of collagen reflect both religious reasons and health issues related to the bovine spongiform encephalopathy outbreak which has led to some consumers needing or desiring alternate sources [275]. Marine gelatins meet many of their concerns. The viscous liquid state of the marine gelatins at room temperature is both a benefit and an issue, good absorption capacity, the survival of enzymatic digestion products in the gastrointestinal tract, enhanced digestibility, and good film-forming properties are the functional properties that mostly are an advantage of marine gelatins in some applications, particularly in biomedical applications and in food uses [272,273,276]. Functional properties of gelatin such as gelling, foaming, stabilizing, or emulsifying ability determine its suitability for many applications in food production, directly or as an ingredient in food coatings, and an active component in packaging material [277]. Some characteristics of marine gelatin, such as gel strength, MW, and melting temperature are lower than those of gelatin from terrestrial species and present a challenge in the industrialization of fish gelatin. Also, there is a difference of gel strength and melting temperatures between cold- and warm-water fish, with gelatin from warm-water fish being more similar to bovine or porcine gelatins [273,278,279]. This is due to the differences in protein characteristics and amino acid composition between cold- and warm-water species as well as a lower hydroxyproline content of cold-water fish [280]. The gel strength of marine-origin gelatin ranges from 98 to 600 g [281]. The composite of a particular gelatin’s properties determines the potential applications. Marine-originated gelatins with lower melting points have some advantages in food application. They can be used for microencapsulation of colorants, enhancement of sensory properties of low-fat foods (flavor), as a food emulsifier, stabilizer, and foaming agent [273,282]. Various approaches have been investigated to improve the functional properties of marine gelatins. Gelatin can be modified using transglutaminase to improve gelatin elasticity and cohesiveness of the gels as well as providing non-thermoreversible gels with lower gel strength [283]. Further, if hydrolyzed with papain, fish gelatin may act as a strong antimicrobial agent [284]. The strength of gelatin gels may be increased by non-electrolytes (glycerol, sorbitol, sucrose) while the gel strength and melting point of fish gelatin can be increased by incorporation of co-enhancers such as Mg, sulphate, sucrose, and transglutaminase [285]. Significant improvement in the gel strength and reduction in viscosity can be obtained using ultraviolet irradiation [278]. Jridi et al. [286] showed that gelatin from cuttlefish stops β-carotene bleaching suggesting its importance in food protection from drying and exposure to light and its possible application in the production of food packaging material. The application of gelatin in food packaging would contribute to reducing drip loss, oxygen-induced changes such as lipid oxidation and color changes. Production of edible coatings that may carry antioxidants and/or antimicrobials might prolong the shelf-life including flavor and odor loss during storage and change the mechanical and barrier properties of the films [287,288]. Gelatin does not meet the dietary requirements for proteins as it is nutritionally unbalanced but when combined with other proteins, the expectation is that it functions like any other protein. It can be used to improve the functional properties of food and beverage products and to enhance the nutritional value of food products.

Besides food applications, the properties of marine gelatin can be improved for other uses as well. The addition of phenolic compounds, e.g., caffeic acid, to gelatin from fish scales enhanced its mechanical biodegradability, and cytocompatible properties, thus making the gelatin more effective material for tissue engineering [289]. After being hydrolyzed with pepsin Pacific cod skin gelatin produced two bioactive peptides that showed a strong inhibitory effect against ACE [247]. This enzyme has an important role in the control of T2DM and hypertension. Although the functional properties of gelatin are species-dependent and vary based on the extraction methods, it is accepted as a non-carcinogenic, economical protein compared to many other proteins but significantly more expensive than bovine and porcine gelatin, and biocompatible for many pharmaceuticals. Different biomedical products of collagen and gelatin such as gels, scaffolds, microspheres, and films have been shown to be usefulness in tissue engineering, implants, and wound dressing [272,290]. 

The use of oral administration, microcryogel injection, and biodegradable scaffolds to promote wound healing at different levels including superficial, deep layer, and systematic levels [272] have been studied. Also, gelatin is used as a wound dressing material and sterile sponge production for medical and dental surgery [291], the main ingredient for both soft and hard gel capsules with an enhance viscosity that prolongs the release from nanoparticles of trapped materials such as drug, vitamins, and minerals [290,292]. More recently, gelatin nanoparticles obtained by nanoprecipitation, were tested for improved mechanical properties such as particle size, shape, and surface chemistry for drug delivery systems [293]. 

## 3. Health Benefit of Nano-Based Materials for Bioactive Compounds from Marine-Based Sources

Nanotechnology is defined as an emerging technology enabling the development of sustainable foods and medicines. For example, fatty acid and amino acid stability in fish meat are improved using nanofibers [294,295]. The intake and stability of vitamins are also important and chitosan-based nanofibers and thymol-loaded electrospun chitosan-based nanofibers could be successfully used to provide nicotinamide acid, pyridoxal, pyridoxine and pyridoxamine in fish fillets [296]. EPA, DHA, and EAA like tryptophan, or B group vitamins are important marine-based compounds for human health [110,297]. The use of probiotic bacteria is important in food systems and for human immune systems. Consumption of fish meat coated with nanoprobiotics (*L. reuteri* and *L. rhamnosus*) could provide a different approach. These nanoprobiotics increased the stability of the fatty acids in fish meat [295,298]. On the other hand, algae are good sources of many essential fatty acids such as ω-3 and ω-6 that are important for the immune system [299]. Also, in medical applications, antidiabetic and anti-inflammatory activities of Au nanoparticles obtained from brown algae seaweed were reported by Venkatraman et al. [300]. 

Nanotechnology applications of marine-based products could be integrated with other food materials having probiotic properties. For example, nanoemulsion technology provided improved physicochemical properties of yogurt enriched with fish oil [301]. Microalgae-based medicines and their biomedical applications could be used to synthesize nano-based materials. For example, nanoparticles with a maximum of 29 and 60 nm diameters obtained from marine fungi like *Aspergillus flavus*, and seagrass like *Cymodocea sp.*, had good activity with anticancer and cytotoxic activities [302,303]. Muthuirulappan and Francis [304] showed that the marine algae product fucoxanthin was safe for humans, and its nanosuspension delivery could enhance the efficacy of supplements. Nanostructures generally provide a larger contact area on the surface of the materials and can provide a good controlled release profile.

Krill are small crustaceans that are particularly abundant in the Northern (Arctic) and Southern (Antarctic) polar seas. Krill oil (KO) is extracted from the Antarctic *Euphausia superba*, the largest of the krill species. Krill is a sustainable source of ω-3 PUFA, including EPA and DHA. While fish oil mainly contains ω-3 PUFA linked to triacylglycerides (TAG), KO contains a significant portion of these FA bound to phospholipids (PL) and phosphatidylcholine (PC) is the most abundant. More than 80% of EPA and DHA in KO are in PC [305,306]. However, it is a relatively high-cost product [307]. Therefore, nanotechnology applications such as nanoparticles (130 nm) may be used to encapsulate KO (*Euphausia superba*) as a dietary supplement [308]. The release profile of KO (1 wt%) from nanoliposomes (217 nm) also showed enhanced oxidative stability. Nanoliposomes could effectively control the release of KO in a simulated gastrointestinal system. These results showed that the nanoform of KO could be used to improve the release profile [309].

## 4. Conclusions

Marine organisms are becoming attractive sources of compounds that show value beyond the nutritional one. New discoveries of diverse biologically active compounds and the growth of their possible applications in food, functional food and supplement development are receiving more attention. Chitosan, chitin, fish oil, EPA and DHA, EAA, peptides, gelatin, polysaccharides, polyphenols, pigments, vitamins, minerals, and other materials have been characterized by their antimicrobial, antioxidant, anti-inflammatory, anti-cancer, anti-tumor, antiviral, antimalarial, anti-obesity, and immunomodulatory properties. Many of these compounds have been used as functional food ingredients or their biological properties have been used to treat/prevent some type of health disorder. They have also been applied in the food industry to enhance the food properties (stabilizer, emulsifier, coating or thickening agent, texture modifier) or to enriched the foods with functional components (ω-3) and allow their application in health-promoting foods for direct consumption.

The global nutraceutical market is rising in constant search of compounds to be applied in the food, beverage and supplement industries. At the same time the consumers are becoming increasingly aware of the role and benefits of the functional foods and supplements on overall health. Both the search for health promoting compounds from natural sources and the development of new functional products has intensified. 

The futures prospective for the use of marine-originated components depend on their bioavailability, development of environmental friendly technologies for their high-yield extractions, preservation from degradation and identification of individual components that can be purified for use in a particular form. The sustainability of marine sources should be considered when they are exploited on the industrial level. Farming and cultivation of the organisms for a particular component and determining the conditions needed for its high yield recovery is a complex undertaking that requires development of new cost-effective technologies. The nanotechnology techniques may be used for the production of nanosystems with marine-based bioactive molecules to protect these functional compounds against degradation. Also, clinical studies are needed, especially for newly discovered compounds to confirm their therapeutic effect, establish their role in health promoting and quantities for daily consummation. These would help the food industry develop functional foods appealing to the consumers. 

## References

[1] Suleria H.A.R., Gobe G., Masci P., Osborne S.A. (2016). Marine Bioactive Compounds and Health Promoting Perspectives; Innovation Pathways for Drug Discovery. Trends Food Sci. Technol..

[2] Mateos R., Pérez-Correa J.R., Domínguez H. (2020). Bioactive Properties of Marine Phenolics. Mar. Drugs.

[3] Ande M.P., Syamala K., SrinivasaRao P., MuraliMohan K., Lingam S.S. (2016). Marine Nutraceuticals. Mar. Omi. Princ. Appl..

[4] (2019). Marine-Derived Drugs Market Growing at a CAGR of 11.20% and Expected to Reach $21,955.6 Million by 2025—Exclusive Report by Infinium Global Research. Infinium Global Research. https://www.medgadget.com/2019/07/marine-derived-drugs-market-growing-at-a-cagr-of-11-20-and-expected-to-reach-21955-6-million-by-2025-exclusive-report-by-infinium-global-research.html.

[5] Sharanagat V.S., Singla V., Singh L., Goyal M.R., Rasul Suleria H.A., Kirubanandan S. (2020). Bioactive Compounds from Marine Sources. Technological Processes for Marine Foods-from Water to Fork: Bioactive Compounds, Industrial Applications and Genomics.

[6] Nalini S., Sandy Richard D., Mohammed Riyaz S.U., Kavitha G., Inbakandan D. (2018). Antibacterial Macro Molecules from Marine Organisms. Int. J. Biol. Macromol..

[7] Bilal M., Iqbal H.M.N. (2020). Biologically Active Macromolecules: Extraction Strategies, Therapeutic Potential and Biomedical Perspective. Int. J. Biol. Macromol..

[8] Sudatta B.P., Sugumar V., Varma R., Nigariga P. (2020). Extraction, Characterization and Antimicrobial Activity of Chitosan from Pen Shell, Pinna Bicolor. Int. J. Biol. Macromol..

[9] Aranaz I., Mengibar M., Harris R., Panos I., Miralles B., Acosta N., Galed G., Heras A. (2009). Functional Characterization of Chitin and Chitosan. Curr. Chem. Biol..

[10] Ngo D.H., Vo T.S., Ngo D.N., Kang K.H., Je J.Y., Pham H.N.D., Byun H.G., Kim S.K. (2015). Biological Effects of Chitosan and Its Derivatives. Food Hydrocoll..

[11] Anraku M., Fujii T., Kondo Y., Kojima E., Hata T., Tabuchi N., Tsuchiya D., Goromaru T., Tsutsumi H., Kadowaki D. (2011). Antioxidant Properties of High Molecular Weight Dietary Chitosan in Vitro and in Vivo. Carbohydr. Polym..

[12] Je J.Y., Park P.J., Kim S.K. (2004). Radical Scavenging Activity of Hetero-Chitooligosaccharides. Eur. Food Res. Technol..

[13] Anraku M., Fujii T., Furutani N., Kadowaki D., Maruyama T., Otagiri M., Gebicki J.M., Tomida H. (2009). Antioxidant Effects of a Dietary Supplement: Reduction of Indices of Oxidative Stress in Normal Subjects by Water-Soluble Chitosan. Food Chem. Toxicol..

[14] Goto M., Iohara D., Michihara A., Ifuku S., Azuma K., Kadowaki D., Maruyama T., Otagiri M., Hirayama F., Anraku M. (2020). Effects of Surface-Deacetylated Chitin Nanofibers on Non-Alcoholic Steatohepatitis Model Rats and Their Gut Microbiota. Int. J. Biol. Macromol..

[15] Ma Z., Garrido-Maestu A., Jeong K.C. (2017). Application, Mode of Action, and in Vivo Activity of Chitosan and Its Micro- and Nanoparticles as Antimicrobial Agents: A Review. Carbohydr. Polym..

[16] Raafat D., Von Bargen K., Haas A., Sahl H.G. (2008). Insights into the Mode of Action of Chitosan as an Antibacterial Compound. Appl. Environ. Microbiol..

[17] Ahmad S.I., Ahmad R., Khan M.S., Kant R., Shahid S., Gautam L., Hasan G.M., Hassan M.I. (2020). Chitin and Its Derivatives: Structural Properties and Biomedical Applications. Int. J. Biol. Macromol..

[18] Chien R.C., Yen M.T., Mau J.L. (2016). Antimicrobial and Antitumor Activities of Chitosan from Shiitake Stipes, Compared to Commercial Chitosan from Crab Shells. Carbohydr. Polym..

[19] Hamed A.A., Abdelhamid I.A., Saad G.R., Elkady N.A., Elsabee M.Z. (2020). Synthesis, Characterization and Antimicrobial Activity of a Novel Chitosan Schiff Bases Based on Heterocyclic Moieties. Int. J. Biol. Macromol..

[20] Bakshi P.S., Selvakumar D., Kadirvelu K., Kumar N.S. (2018). Comparative Study on Antimicrobial Activity and Biocompatibility of N-Selective Chitosan Derivatives. React. Funct. Polym..

[21] Salama H.E., Abdel Aziz M.S., Sabaa M.W. (2019). Development of Antibacterial Carboxymethyl Cellulose/Chitosan Biguanidine Hydrochloride Edible Films Activated with Frankincense Essential Oil. Int. J. Biol. Macromol..

[22] Muanprasat C., Chatsudthipong V. (2017). Chitosan Oligosaccharide: Biological Activities and Potential Therapeutic Applications. Pharmacol. Ther..

[23] Huang R., Mendis E., Kim S.K. (2005). Improvement of ACE Inhibitory Activity of Chitooligosaccharides (COS) by Carboxyl Modification. Bioorg. Med. Chem..

[24] Park P.J., Ahn C.B., Jeon Y.J., Je J.Y. (2008). Renin Inhibition Activity by Chitooligosaccharides. Bioorganic Med. Chem. Lett..

[25] Vo T.S., Kong C.S., Kim S.K. (2011). Inhibitory Effects of Chitooligosaccharides on Degranulation and Cytokine Generation in Rat Basophilic Leukemia RBL-2H3 Cells. Carbohydr. Polym..

[26] Chung M.J., Park J.K., Park Y.I. (2012). Anti-Inflammatory Effects of Low-Molecular Weight Chitosan Oligosaccharides in IgE-Antigen Complex-Stimulated RBL-2H3 Cells and Asthma Model Mice. Int. Immunopharmacol..

[27] Hu X., Tao N., Wang X., Xiao J., Wang M. (2016). Marine-Derived Bioactive Compounds with Anti-Obesity Effect: A Review. J. Funct. Foods.

[28] Chiu C.Y., Chang T.C., Liu S.H., Chiang M.T. (2017). The Regulatory Effects of Fish Oil and Chitosan on Hepatic Lipogenic Signals in High-Fat Diet-Induced Obese Rats. J. Food Drug Anal..

[29] Inanli A.G., Tümerkan E.T.A., El Abed N., Regenstein J.M., Özogul F. (2020). The Impact of Chitosan on Seafood Quality and Human Health: A Review. Trends Food Sci. Technol..

[30] Panith N., Wichaphon J., Lertsiri S., Niamsiri N. (2016). Effect of Physical and Physicochemical Characteristics of Chitosan on Fat-Binding Capacities under in Vitro Gastrointestinal Conditions. LWT Food Sci. Technol..

[31] Wydro P., Krajewska B., Ha K., Wydro P. (2007). Chitosan as a Lipid Binder: A Langmuir Monolayer Study of Chitosan−Lipid Interactions Chitosan as a Lipid Binder: A Langmuir Monolayer Study of Chitosan-Lipid Interactions. Am. Chem. Soc..

[32] Anraku M., Gebicki J.M., Iohara D., Tomida H., Uekama K., Maruyama T., Hirayama F., Otagiri M. (2018). Antioxidant Activities of Chitosans and Its Derivatives in in Vitro and in Vivo Studies. Carbohydr. Polym..

[33] Azuma K., Ifuku S., Osaki T., Okamoto Y., Minami S. (2014). Preparation and Biomedical Applications of Chitin and Chitosan Nanofibers. J. Biomed. Nanotechnol..

[34] Bondiolotti G., Bareggi S.R., Frega N.G., Strabioli S., Cornelli U. (2007). Activity of Two Different Polyglucosamines, L112^®^ and FF45^®^, on Body Weight in Male Rats. Eur. J. Pharmacol..

[35] Kaats G.R., Michalek J.E., Preuss H.G. (2006). Evaluating Efficacy of a Chitosan Product Using a Double-Blinded, Placebo-Controlled Protocol. J. Am. Coll. Nutr..

[36] Gades M.D., Stern J.S. (2003). Chitosan Supplementation and Fecal Fat Excretion in Men. Obes. Res..

[37] Hayashi K., Ito M. (2002). Antidiabetic Action of Low Molecular Weight Chitosan in Genetically Obese Diabetic KK-Ay Mice. Biol. Pharm. Bull..

[38] Gorzelanny C., Pöppelmann B., Strozyk E., Moerschbacher B.M., Schneider S.W. (2007). Specific Interaction between Chitosan and Matrix Metalloprotease 2 Decreases the Invasive Activity of Human Melanoma Cells. Biomacromolecules.

[39] Sayari N., Sila A., Abdelmalek B.E., Abdallah R.B., Ellouz-Chaabouni S., Bougatef A., Balti R. (2016). Chitin and Chitosan from the Norway Lobster By-Products: Antimicrobial and Anti-Proliferative Activities. Int. J. Biol. Macromol..

[40] Resmi R., Yoonus J., Beena B. (2020). Anticancer and Antibacterial Activity of Chitosan Extracted from Shrimp Shell Waste. Mater. Today Proc..

[41] El-Naggar M.M., Haneen D.S.A., Mehany A.B.M., Khalil M.T. (2020). New Synthetic Chitosan Hybrids Bearing Some Heterocyclic Moieties with Potential Activity as Anticancer and Apoptosis Inducers. Int. J. Biol. Macromol..

[42] Sedghi R., Gholami M., Shaabani A., Saber M., Niknejad H. (2020). Preparation of Novel Chitosan Derivative Nanofibers for Prevention of Breast Cancer Recurrence. Eur. Polym. J..

[43] Wang H.M.D., Li X.C., Lee D.J., Chang J.S. (2017). Potential Biomedical Applications of Marine Algae. Bioresour. Technol..

[44] Zhao C., Yang C., Liu B., Lin L., Sarker S.D., Nahar L., Yu H., Cao H., Xiao J. (2018). Bioactive Compounds from Marine Macroalgae and Their Hypoglycemic Benefits. Trends Food Sci. Technol..

[45] Khanna P., Kaur A., Goyal D. (2019). Algae-Based Metallic Nanoparticles: Synthesis, Characterization and Applications. J. Microbiol. Methods.

[46] Mekinić I.G., Skroza D., Šimat V., Hamed I., Čagalj M., Perković Z.P. (2019). Phenolic Content of Brown Algae (Pheophyceae) Species: Extraction, Identification, and Quantification. Biomolecules.

[47] Pangestuti R., Kim S.K. (2011). Biological Activities and Health Benefit Effects of Natural Pigments Derived from Marine Algae. J. Funct. Foods.

[48] Aguirre-Joya J.A., Chacón-Garza L.E., Valdivia-Najár G., Arredondo-Valdés R., Castro-López C., Ventura-Sobrevilla J.M., Aguilar-Gonzáles C.N., Boone-Villa D. (2020). Nanosystems of Plant-Based Pigments and Its Relationship with Oxidative Stress. Food Chem. Toxicol..

[49] Rengasamy K.R., Mahomoodally M.F., Aumeeruddy M.Z., Zengin G., Xiao J., Kim D.H. (2020). Bioactive Compounds in Seaweeds: An Overview of Their Biological Properties and Safety. Food Chem. Toxicol..

[50] Chakdar H., Pabbi S. (2017). Algal Pigments for Human Health and Cosmeceuticals.

[51] Martins N., Ferreira I.C.F.R. (2017). Wastes and By-Products: Upcoming Sources of Carotenoids for Biotechnological Purposes and Health-Related Applications. Trends Food Sci. Technol..

[52] Ambati R.R., Gogisetty D., Aswathanarayana R.G., Ravi S., Bikkina P.N., Bo L., Yuepeng S. (2019). Industrial Potential of Carotenoid Pigments from Microalgae: Current Trends and Future Prospects. Crit. Rev. Food Sci. Nutr..

[53] Ngamwonglumlert L., Devahastin S., Melton L., Shahidi F., Varelis P. (2019). Carotenoids. Encyclopedia of Food Chemistry.

[54] Nagappan H., Pee P.P., Kee S.H.Y., Ow J.T., Yan S.W., Chew L.Y., Kong K.W. (2017). Malaysian Brown Seaweeds Sargassum Siliquosum and Sargassum Polycystum: Low Density Lipoprotein (LDL) Oxidation, Angiotensin Converting Enzyme (ACE), α-Amylase, and α-Glucosidase Inhibition Activities. Food Res. Int..

[55] Ganesan A.R., Tiwari U., Rajauria G. (2019). Seaweed Nutraceuticals and Their Therapeutic Role in Disease Prevention. Food Sci. Hum. Wellness.

[56] Kraan S. (2013). Pigments and Minor Compounds in Algae.

[57] Biris-Dorhoi E.S., Michiu D., Pop C.R., Rotar A.M., Tofana M., Pop O.L., Socaci S.A., Farcas A.C. (2020). Macroalgae—A Sustainable Source of Chemical Compounds with Biological Activities. Nutrients.

[58] Thakur M. (2020). Marine Bioactive Components: Sources, Health Benefits, and Future Prospects. Technological Processes for Marine Foods-from Water to Fork: Bioactive Compounds, Industrial Applications and Genomics.

[59] Sanjeewa K.K.A., Kang N., Ahn G., Jee Y., Kim Y.T., Jeon Y.J. (2018). Bioactive Potentials of Sulfated Polysaccharides Isolated from Brown Seaweed Sargassum Spp in Related to Human Health Applications: A Review. Food Hydrocoll..

[60] Udayangani R.M.A.C., Somasiri G.D.P., Wickramasinghe I., Kim S. (2020). Potential Health Benefits of Sulfated Polysaccharides from Marine Algae. Encycl. Mar. Biotechnol..

[61] Sanjeewa K.K.A., Lee J.S., Kim W.S., Jeon Y.J. (2017). The Potential of Brown-Algae Polysaccharides for the Development of Anticancer Agents: An Update on Anticancer Effects Reported for Fucoidan and Laminaran. Carbohydr. Polym..

[62] Fernando I.P.S., Sanjeewa K.K.A., Samarakoon K.W., Lee W.W., Kim H.S., Kang N., Ranasinghe P., Lee H.S., Jeon Y.J. (2017). A Fucoidan Fraction Purified from Chnoospora Minima; a Potential Inhibitor of LPS-Induced Inflammatory Responses. Int. J. Biol. Macromol..

[63] Fitton J.H., Stringer D.N., Park A.Y., Karpiniec S.S. (2019). Therapies from Fucoidan: New Developments. Mar. Drugs.

[64] Han M.H., Lee D.S., Jeong J.W., Hong S.H., Choi I.W., Cha H.J., Kim S., Kim H.S., Park C., Kim G.Y. (2017). Fucoidan Induces ROS-Dependent Apoptosis in 5637 Human Bladder Cancer Cells by Downregulating Telomerase Activity via Inactivation of the PI3K/Akt Signaling Pathway. Drug Dev. Res..

[65] Palanisamy S., Vinosha M., Manikandakrishnan M., Anjali R., Rajasekar P., Marudhupandi T., Manikandan R., Vaseeharan B., Prabhu N.M. (2018). Investigation of Antioxidant and Anticancer Potential of Fucoidan from Sargassum Polycystum. Int. J. Biol. Macromol..

[66] Narayani S.S., Saravanan S., Ravindran J., Ramasamy M.S., Chitra J. (2019). In Vitro Anticancer Activity of Fucoidan Extracted from Sargassum Cinereum against Caco-2 Cells. Int. J. Biol. Macromol..

[67] Ermakova S., Sokolova R., Kim S.M., Um B.H., Isakov V., Zvyagintseva T. (2011). Fucoidans from Brown Seaweeds Sargassum Hornery, Eclonia Cava, Costaria Costata: Structural Characteristics and Anticancer Activity. Appl. Biochem. Biotechnol..

[68] Kadam S.U., Tiwari B.K., O’Donnell C.P. (2015). Extraction, Structure and Biofunctional Activities of Laminarin from Brown Algae. Int. J. Food Sci. Technol..

[69] Zargarzadeh M., Amaral A.J.R., Custódio C.A., Mano J.F. (2020). Biomedical Applications of Laminarin. Carbohydr. Polym..

[70] Sellimi S., Maalej H., Rekik D.M., Benslima A., Ksouda G., Hamdi M., Sahnoun Z., Li S., Nasri M., Hajji M. (2018). Antioxidant, Antibacterial and in Vivo Wound Healing Properties of Laminaran Purified from Cystoseira Barbata Seaweed. Int. J. Biol. Macromol..

[71] Custódio C.A., Reis R.L., Mano J.F. (2016). Photo-Cross-Linked Laminarin-Based Hydrogels for Biomedical Applications. Biomacromolecules.

[72] Fertah M., Belfkira A., Dahmane E.M., Taourirte M., Brouillette F. (2017). Extraction and Characterization of Sodium Alginate from Moroccan Laminaria Digitata Brown Seaweed. Arab. J. Chem..

[73] Draget K.I., Taylor C. (2011). Chemical, Physical and Biological Properties of Alginates and Their Biomedical Implications. Food Hydrocoll..

[74] Emerton V., Choi E. (2008). Essential Guide to Food Additives.

[75] Adrian G., Mihai M., Vodnar D.C. (2019). The use of chitosan, alginate, and pectin in the biomedical and food sector—Biocompatibility, bioadhesiveness, and biodegradability. Polymers (Basel).

[76] Andryukov B.G., Besednova N.N., Kuznetsova T.A., Zaporozhets T.S., Ermakova S.P., Zvyagintseva T.N., Chingizova E.A., Gazha A.K., Smolina T.P. (2020). Sulfated Polysaccharides from Marine Algae as a Basis of Modern Biotechnologies for Creating Wound Dressings: Current Achievements and Future Prospects. Biomedicines.

[77] Qureshi D., Nayak S.K., Maji S., Kim D., Banerjee I., Pal K. (2019). Carrageenan: A Wonder Polymer from Marine Algae for Potential Drug Delivery Applications. Curr. Pharm. Des..

[78] Yegappan R., Selvaprithiviraj V., Amirthalingam S., Jayakumar R. (2018). Carrageenan Based Hydrogels for Drug Delivery, Tissue Engineering and Wound Healing. Carbohydr. Polym..

[79] Besednova N.N., Zaporozhets T.S., Kuznetsova T.A., Makarenkova I.D., Kryzhanovsky S.P., Fedyanina L.N., Ermakova S.P. (2020). Extracts and Marine Algae Polysaccharides in Therapy and Prevention of Inflammatory Diseases of the Intestine. Mar. Drugs.

[80] Lahaye M., Robic A. (2007). Structure and Function Properties of Ulvan, a Polysaccharide from Green Seaweeds. Biomacromolecules.

[81] Kidgell J.T., Magnusson M., de Nys R., Glasson C.R.K. (2019). Ulvan: A Systematic Review of Extraction, Composition and Function. Algal Res..

[82] Cunha L., Grenha A. (2016). Sulfated Seaweed Polysaccharides as Multifunctional Materials in Drug Delivery Applications. Mar. Drugs.

[83] Abd-Ellatef G.E.F., Ahmed O.M., Abdel-Reheim E.S., Abdel-Hamid A.H.Z. (2017). Ulva Lactuca Polysaccharides Prevent Wistar Rat Breast Carcinogenesis through the Augmentation of Apoptosis, Enhancement of Antioxidant Defense System, and Suppression of Inflammation. Breast Cancer Targets Ther..

[84] Naczk M., Shahidi F. (2004). Phenolics in Food and Nutraceuticals.

[85] Kirke D.A., Rai D.K., Smyth T.J., Stengel D.B. (2019). An Assessment of Temporal Variation in the Low Molecular Weight Phlorotannin Profiles in Four Intertidal Brown Macroalgae. Algal Res..

[86] Chen Y., Lin H., Li Z., Mou Q. (2015). The Anti-Allergic Activity of Polyphenol Extracted from Five Marine Algae. J. Ocean Univ. China.

[87] Pangestuti R., Kim S.K. (2011). Neuroprotective Effects of Marine Algae. Mar. Drugs.

[88] Gómez-Guzmán M., Rodríguez-Nogales A., Algieri F., Gálvez J. (2018). Potential Role of Seaweed Polyphenols in Cardiovascular-Associated Disorders. Mar. Drugs.

[89] Nwosu F., Morris J., Lund V.A., Stewart D., Ross H.A., McDougall G.J. (2011). Anti-Proliferative and Potential Anti-Diabetic Effects of Phenolic-Rich Extracts from Edible Marine Algae. Food Chem..

[90] Liu H.C., Chang C.J., Yang T.H., Chiang M.T. (2017). Long-Term Feeding of Red Algae (Gelidium Amansii) Ameliorates Glucose and Lipid Metabolism in a High Fructose Diet-Impaired Glucose Tolerance Rat Model. J. Food Drug Anal..

[91] Yoshinaga K., Nakai Y., Izumi H., Nagaosa K., Ishijima T., Nakano T., Abe K. (2018). Oral Administration of Edible Seaweed Undaria Pinnatifida (Wakame) Modifies Glucose and Lipid Metabolism in Rats: A DNA Microarray Analysis. Mol. Nutr. Food Res..

[92] Vestland T.L., Jacobsen Ø., Sande S.A., Myrset A.H., Klaveness J. (2016). Characterization of Omega-3 Tablets. Food Chem..

[93] Schmidt N., Møller G., Bæksgaard L., Østerlind K., Stark K.D., Lauritzen L., Andersen J.R. (2020). Fish Oil Supplementation in Cancer Patients. Capsules or Nutritional Drink Supplements? A Controlled Study of Compliance. Clin. Nutr. ESPEN.

[94] Šimat V., Soldo B., Skroza D., Ljubenkov I., Generalić Mekinić I. (2019). Production and Refinement of Omega-3 Rich Oils from Processing By-Products of Farmed Fish Species. Foods.

[95] Jamshidi A., Cao H., Xiao J., Simal-Gandara J. (2020). Advantages of Techniques to Fortify Food Products with the Benefits of Fish Oil. Food Res. Int..

[96] Naqshbandi A., Khan M.W., Rizwan S., ur Rehman S., Khan F. (2012). Studies on the Protective Effect of Dietary Fish Oil on Cisplatin Induced Nephrotoxicity in Rats. Food Chem. Toxicol..

[97] Das S., Paul B., Sengupta J., Datta A. (2009). Beneficial Effects of Fish Oil to Human Health: A Review. Agric. Rev..

[98] Salgado P.R., Di Giorgio L., Musso Y.S., Mauri A.N. (2018). Bioactive Packaging: Combining Nanotechnologies with Packaging for Improved Food Functionality.

[99] Farooqui A.A. (2009). Beneficial Effects of Fish Oil on Human Brain.

[100] Pipingas A., Sinclair A., Croft K.D., Januszewski A.S., Jenkins A.J., Mori T.A., Cockerell R., Grima N.A., Stough C., Scholey A. (2015). Fish Oil and Multivitamin Supplementation Reduces Oxidative Stress but Not Inflammation in Healthy Older Adults: A Randomised Controlled Trial. J. Funct. Foods.

[101] Curado Borges M., de Miranda Moura dos Santos F., Weiss Telles R., Melo de Andrade M.V., Toulson Davisson Correia M.I., Lanna C.C.D. (2017). Omega-3 Fatty Acids, Inflammatory Status and Biochemical Markers of Patients with Systemic Lupus Erythematosus: A Pilot Study. Rev. Bras. Reumatol..

[102] De Souza D.R., da Silva Pieri B.L., Comim V.H., de Oliveira Marques S., Luciano T.F., Rodrigues M.S., De Souza C.T. (2020). Fish Oil Reduces Subclinical Inflammation, Insulin Resistance, and Atherogenic Factors in Overweight/Obese Type 2 Diabetes Mellitus Patients: A Pre-Post Pilot Study. J. Diabetes Complicat..

[103] Burri L., Hoem N., Banni S., Berge K. (2012). Marine Omega-3 Phospholipids: Metabolism and Biological Activities. Int. J. Mol. Sci..

[104] Xie D., Gong M., Wei W., Jin J., Wang X., Wang X., Jin Q. (2019). Antarctic Krill (*Euphausia superba*) Oil: A Comprehensive Review of Chemical Composition, Extraction Technologies, Health Benefits, and Current Applications. Compr. Rev. Food Sci. Food Saf..

[105] Suseno S.H., Saraswati S., Hayati S., Izaki A.F. (2014). Fatty Acid Composition of Some Potential Fish Oil from Production Centers in Indonesia. Orient. J. Chem..

[106] Hamed I., Özogul F., Özogul Y., Regenstein J.M. (2015). Marine Bioactive Compounds and Their Health Benefits: A Review. Compr. Rev. Food Sci. Food Saf..

[107] DiNicolantonio J.J., O’Keefe J.H. (2017). Good Fats versus Bad Fats: A Comparison of Fatty Acids in the Promotion of Insulin Resistance, Inflammation, and Obesity. Mo. Med..

[108] Huang T.H., Wang P.W., Yang S.C., Chou W.L., Fang J.Y. (2018). Cosmetic and Therapeutic Applications of Fish Oil’s Fatty Acids on the Skin. Mar. Drugs.

[109] Alaswad K., Lavie C.J., Milani R.V., O’Keefe J.H. (2002). Fish Oil in Cardiovascular Prevention. Ochsner J..

[110] Kromhout D., Yasuda S., Geleijnse J.M., Shimokawa H. (2012). Fish Oil and Omega-3 Fatty Acids in Cardiovascular Disease: Do They Really Work?. Eur. Heart J..

[111] Connor W.E., Cefrancesco C.A., Connor S. (1993). N-3 Fatty Acids from Fish Oil Effects on Plasma Lipoproteins and Hypertriglyceridemic Patients. Ann. N. Y. Acad. Sci..

[112] Sun T., Xu Z., Prinyawiwatkul W. (2006). FA Composition of the Oil Extracted from Farmed Atlantic Salmon (Salmo Salar L.) Viscera. JAOCS J. Am. Oil Chem. Soc..

[113] Choulis N.H. (2011). Miscellaneous Drugs Materials, Medical Devices, and Techniques. Side Effects of Drugs Annual.

[114] Kahveci D., Falkeborg M., Gregersen S., Xu X. (2014). Upgrading of Farmed Salmon Oil Through Lipase-Catalyzed Hydrolysis. Open Biotechnol. J..

[115] Uçak I., Oz M., Maqsood S. (2019). Products Based on Omega-3 Polyunsaturated Fatty Acids and Health Effects. The Role of Alternative and Innovative Food Ingredients and Products in Consumer Wellness.

[116] Haq M., Park S.K., Kim M.J., Cho Y.J., Chun B.S. (2018). Modifications of Atlantic Salmon By-Product Oil for Obtaining Different ω-3 Polyunsaturated Fatty Acids Concentrates: An Approach to Comparative Analysis. J. Food Drug Anal..

[117] Toyoshima K., Noguchi R., Hosokawa M., Fukunaga K., Nishiyama T., Takahashi R., Miyashita K. (2004). Separation of Sardine Oil without Heating from Surimi Waste and Its Effect on Lipid Metabolism in Rats. J. Agric. Food Chem..

[118] Solaesa Á.G., Bucio S.L., Sanz M.T., Beltrán S., Rebolleda S. (2014). Characterization of Triacylglycerol Composition of Fish Oils by Using Chromatographic Techniques. J. Oleo Sci..

[119] Sharma R., Katz J. (2013). Fish Proteins in Coronary Artery Disease Prevention: Amino Acid–Fatty Acid Concept. Bioactive Food as Dietary Interventions for Cardiovascular Disease.

[120] Aidos I., Van Der Padt A., Boom R.M., Luten J.B. (2003). Quality of Crude Fish Oil Extracted from Herring Byproducts of Varying States of Freshness. J. Food Sci..

[121] Kim Y.G., Lee J.H., Raorane C.J., Oh S.T., Park J.G., Lee J. (2018). Herring Oil and Omega Fatty Acids Inhibit Staphylococcus Aureus Biofilm Formation and Virulence. Front. Microbiol..

[122] Shireman R., Caballero B. (2003). Essential Fatty Acids. Encyclopedia of Food Sciences and Nutrition.

[123] Alexa-Stratulat T., Luca A., Badescu M., Bohotin C.R., Alexa I.D. (2017). Nutritional Modulators in Chemotherapy-Induced Neuropathic Pain. Nutritional Modulators of Pain in the Aging Population.

[124] Hahn B.H., Kono D.H., Wallace D.J., Hahn B.H. (2019). Animal Models in Lupus. Dubois’ Lupus Erythematosus and Related Syndromes.

[125] Pigott G.M., Tucker B.W. (2003). Production Composition and Properties Dietary Importance Production. Encyclopedia of Food Sciences and Nutrition.

[126] Shah M.A., Niaz K., Aslam N., Vargas-de la Cruz C., Kabir A., Khan A.H., Khan F., Panichayupakaranant P. (2020). Analysis of Proteins, Peptides, and Amino Acids. Recent Advances in Natural Products Analysis.

[127] Harnedy P.A., FitzGerald R.J. (2012). Bioactive Peptides from Marine Processing Waste and Shellfish: A Review. J. Funct. Foods.

[128] Rogers M., Bare R., Gray A., Scott-Moelder T., Heintz R. (2019). Assessment of Two Feeds on Survival, Proximate Composition, and Amino Acid Carbon Isotope Discrimination in Hatchery-Reared Chinook Salmon. Fish. Res..

[129] Özogul F., Hamed I., Özogul Y., Regenstein J.M. (2019). Crustacean By-Products. Encyclopedia of Food Chemistry.

[130] Pereira D.M., Valentão P., Teixeira N., Andrade P.B. (2013). Amino Acids, Fatty Acids and Sterols Profile of Some Marine Organisms from Portuguese Waters. Food Chem..

[131] Chandika P., Ko S.C., Jung W.K. (2015). Marine-Derived Biological Macromolecule-Based Biomaterials for Wound Healing and Skin Tissue Regeneration. Int. J. Biol. Macromol..

[132] Shiels K., Murray P., Saha S.K. (2019). Marine Cyanobacteria as Potential Alternative Source for GABA Production. Bioresour. Technol. Rep..

[133] Lee S.Y., Hur S.J. (2017). Antihypertensive Peptides from Animal Products, Marine Organisms, and Plants. Food Chem..

[134] Mohanty B., Mahanty A., Ganguly S., Sankar T.V., Chakraborty K., Rangasamy A., Paul B., Sarma D., Mathew S., Asha K.K. (2014). Amino Acid Compositions of 27 Food Fishes and Their Importance in Clinical Nutrition. J. Amino Acids.

[135] Qi C., Wang X., Han F., Jia Y., Lin Z., Wang C., Lu J., Yang L., Wang X., Li E. (2019). Arginine Supplementation Improves Growth, Antioxidant Capacity, Immunity and Disease Resistance of Juvenile Chinese Mitten Crab, Eriocheir Sinensis. Fish Shellfish Immunol..

[136] Flores E., Arévalo S., Burnat M. (2019). Cyanophycin and Arginine Metabolism in Cyanobacteria. Algal Res..

[137] Pyz-Łukasik R., Paszkiewicz W. (2018). Species Variations in the Proximate Composition, Amino Acid Profile, and Protein Quality of the Muscle Tissue of Grass Carp, Bighead Carp, Siberian Sturgeon, and Wels Catfish. J. Food Qual..

[138] Narayanasamy A., Balde A., Raghavender P., Shashanth D., Abraham J., Joshi I., Nazeer R.A. (2020). Isolation of Marine Crab (Charybdis Natator) Leg Muscle Peptide and Its Anti-Inflammatory Effects on Macrophage Cells. Biocatal. Agric. Biotechnol..

[139] Holeček M. (2020). Histidine in Health and Disease: Metabolism, Physiological Importance, and Use as a Supplement. Nutrients.

[140] Wu G. (2013). Functional Amino Acids in Nutrition and Health. Amino Acids.

[141] Tabakaeva O.V., Tabakaev A.V., Piekoszewski W. (2018). Nutritional Composition and Total Collagen Content of Two Commercially Important Edible Bivalve Molluscs from the Sea of Japan Coast. J. Food Sci. Technol..

[142] Cutrona K.J., Kaufman B.A., Figueroa D.M., Elmore D.E. (2015). Role of Arginine and Lysine in the Antimicrobial Mechanism of Histone-Derived Antimicrobial Peptides. FEBS Lett..

[143] Olin-Sandoval V., Yu J.S.L., Miller-Fleming L., Alam M.T., Kamrad S., Correia-Melo C., Haas R., Segal J., Peña Navarro D.A., Herrera-Dominguez L. (2019). Lysine Harvesting Is an Antioxidant Strategy and Triggers Underground Polyamine Metabolism. Nature.

[144] Bemani E., Ghanati F., Rezaei A., Jamshidi M. (2013). Effect of Phenylalanine on Taxol Production and Antioxidant Activity of Extracts of Suspension-Cultured Hazel (Corylus Avellana L.) Cells. J. Nat. Med..

[145] Hasegawa S., Ichiyama T., Sonaka I., Ohsaki A., Okada S., Wakiguchi H., Kudo K., Kittaka S., Hara M., Furukawa S. (2012). Cysteine, Histidine and Glycine Exhibit Anti-Inflammatory Effects in Human Coronary Arterial Endothelial Cells. Clin. Exp. Immunol..

[146] Wang W., Wu Z., Dai Z., Yang Y., Wang J., Wu G. (2013). Glycine Metabolism in Animals and Humans: Implications for Nutrition and Health. Amino Acids.

[147] Khalili Tilami S., Sampels S. (2018). Nutritional Value of Fish: Lipids, Proteins, Vitamins, and Minerals. Rev. Fish. Sci. Aquac..

[148] Pal J., Shukla B.N., Maurya A.K., Verma H.O. (2018). A Review on Role of Fish in Human Nutrition with Special Emphasis to Essential Fatty Acid. Int. J. Fish. Acquat. Stud..

[149] Bruno S.F., Ekorong F.J.A.A., Karkal S.S., Cathrine M.S.B., Kudre T.G. (2019). Green and Innovative Techniques for Recovery of Valuable Compounds from Seafood By-Products and Discards: A Review. Trends Food Sci. Technol..

[150] Owuamanam S., Cree D. (2020). Progress of Bio-Calcium Carbonate Waste Eggshell and Seashell Fillers in Polymer Composites: A Review. J. Compos. Sci..

[151] Menon V.V., Lele S.S., Kim S.-K. (2015). Nutraceuticals and Bioactive Compounds from Seafood Processing Waste. Springer Handbook of Marine Biotechnology.

[152] Paradelo R., Conde-Cid M., Cutillas-Barreiro L., Arias-Estévez M., Nóvoa-Muñoz J.C., Álvarez-Rodríguez E., Fernández-Sanjurjo M.J., Núñez-Delgado A. (2016). Phosphorus Removal from Wastewater Using Mussel Shell: Investigation on Retention Mechanisms. Ecol. Eng..

[153] Zhang L., Zhang C., Zhang R., Jiang D., Zhu Q., Wang S. (2019). Extraction and Characterization of HA/β-TCP Biphasic Calcium Phosphate from Marine Fish. Mater. Lett..

[154] Miranda G., Sousa F., Costa M.M., Bartolomeu F., Silva F.S., Carvalho O. (2019). Surface Design Using Laser Technology for Ti6Al4V-Hydroxyapatite Implants. Opt. Laser Technol..

[155] Antoniac I.V., Filipescu M., Barbaro K., Bonciu A., Birjega R., Cotrut C.M., Galvano E., Fosca M., Fadeeva I.V., Vadalà G. (2020). Iron Ion-Doped Tricalcium Phosphate Coatings Improve the Properties of Biodegradable Magnesium Alloys for Biomedical Implant Application. Adv. Mater. Interfaces.

[156] Castelo-Branco C., Cancelo Hidalgo M.J., Palacios S., Ciria-Recasens M., Fernández-Pareja A., Carbonell-Abella C., Manasanch J., Haya-Palazuelos J. (2020). Efficacy and Safety of Ossein-Hydroxyapatite Complex versus Calcium Carbonate to Prevent Bone Loss. Climacteric.

[157] Hanh N.T., Bich P.T.N., Thao H.T.T. (2019). Acute and Subchronic Oral Toxicity Assessment of Calcium Hydroxyapatite-Alginate in Animals. Vietnam J. Chem..

[158] Remya N.S., Syama S., Sabareeswaran A., Mohanan P.V. (2017). Investigation of Chronic Toxicity of Hydroxyapatite Nanoparticles Administered Orally for One Year in Wistar Rats.E. Mater. Sci. Eng. C.

[159] Suresh P.V., Kudre T.G., Johny L.C. (2018). Sustainable Valorization of Seafood Processing By-Product/Discard. Waste to Wealth.

[160] Bubel F., Dobrzański Z., Bykowski P.J., Chojnacka K., Opaliński S., Trziszka T. (2015). Production of Calcium Preparations by Technology of Saltwater Fish by Product Processing. Open Chem..

[161] Nemati M., Huda N., Ariffin F. (2017). Development of Calcium Supplement from Fish Bone Wastes of Yellowfin Tuna (Thunnus Albacares) and Characterization of Nutritional Quality. Int. Food Res. J..

[162] Flammini L., Martuzzi F., Vivo V., Ghirri A., Salomi E., Bignetti E., Barocelli E. (2016). Hake Fish Bone as a Calcium Source for Efficient Bone Mineralization. Int. J. Food Sci. Nutr..

[163] Yin T., Du H., Zhang J., Xiong S. (2016). Preparation and Characterization of Ultrafine Fish Bone Powder. J. Aquat. Food Prod. Technol..

[164] Guéguen L., Pointillart A. (2000). The Bioavailability of Dietary Calcium. J. Am. Coll. Nutr..

[165] Li J., Yin T., Xiong S., Huang Q., You J., Hu Y., Liu R., Li Y.J. (2020). Mechanism on Releasing and Solubilizing of Fish Bone Calcium during Nano-Milling. J. Food Process Eng..

[166] Yin T., Park J.W., Xiong S. (2015). Physicochemical Properties of Nano Fish Bone Prepared by Wet Media Milling. LWT Food Sci. Technol..

[167] Huang S., Chen J.C., Hsu C.W., Chang W.H. (2009). Effects of Nano Calcium Carbonate and Nano Calcium Citrate on Toxicity in ICR Mice and on Bone Mineral Density in an Ovariectomized Mice Model. Nanotechnology.

[168] Javeed A., Mahendrakar N.S. (1995). Effect of Different Levels of Molasses and Salt on Acid Production and Volume of Fermenting Mass During Ensiling of Tropical Freshwater Fish Viscera. J. Food Sci. Technol..

[169] Giri S.S., Sahoo S.K., Sahu A.K., Mukhopadhyay P.K. (2000). Nutrient Digestibility and Intestinal Enzyme Activity of Clarias Batrachus (Linn.) Juveniles Fed on Dried Fish and Chicken Viscera Incorporated Diets. Bioresour. Technol..

[170] Kandyliari A., Mallouchos A., Papandroulakis N., Golla J.P., Lam T.K.T., Sakellari A., Karavoltsos S., Vasiliou V., Kapsokefalou M. (2020). Nutrient Composition and Fatty Acid and Protein Profiles of Selected Fish By-Products. Foods.

[171] Afonso C., Bandarra N.M., Nunes L., Cardoso C. (2016). Tocopherols in Seafood and Aquaculture Products. Crit. Rev. Food Sci. Nutr..

[172] Laskowski W., Górska-Warsewicz H., Kulykovets O. (2018). Meat, Meat Products and Seafood as Sources of Energy and Nutrients in the Average Polish Diet. Nutrients.

[173] Wells M.L., Potin P., Craigie J.S., Raven J.A., Merchant S.S., Helliwell K.E., Smith A.G., Camire M.E., Brawley S.H. (2017). Algae as Nutritional and Functional Food Sources: Revisiting Our Understanding. J. Appl. Phycol..

[174] Hosomi R., Yoshida M., Fukunaga K. (2012). Seafood Consumption and Components for Health. Glob. J. Health Sci..

[175] Lund E.K. (2013). Health Benefits of Seafood; Is It Just the Fatty Acids?. Food Chem..

[176] Nadeeshani H., Rajapakse N., Kim S. (2020). Traditional and Novel Seafood Processing Techniques Targeting Human Health Promotion. Encyclopedia of Marine Biotechnology.

[177] Šimat V., Vlahović J., Soldo B., Generalić Mekinić I., Čagalj M., Hamed I., Skroza D. (2020). Production and Characterization of Crude Oils from Seafood Processing By-Products. Food Biosci..

[178] Nurjanah, Nurilmala M., Hidayat T., Sudirdjo F. (2016). Characteristics of Seaweed as Raw Materials for Cosmetics. Aquat. Procedia.

[179] Araújo M., Alves R.C., Pimentel F.B., Costa A.S.G., Fernandes T.J.R., Valente L.M.P., Rema P., Oliveira M.B.P.P. (2015). New Approach for Vitamin E Extraction in Rainbow Trout Flesh: Application in Fish Fed Commercial and Red Seaweed-Supplemented Diets. Eur. J. Lipid Sci. Technol..

[180] Graff I.E., Øyen J., Kjellevold M., Frøyland L., Gjesdal C.G., Almås B., Rosenlund G., Lie Ø. (2016). Reduced Bone Resorption by Intake of Dietary Vitamin D and K from Tailor-Made Atlantic Salmon: A Randomized Intervention Trial. Oncotarget.

[181] Scurria A., Lino C., Pitonzo R., Pagliaro M., Avellone G., Ciriminna R. (2020). Vitamin D3 in Fish Oil Extracted with Limonene from Anchovy Leftovers. Chem. Data Collect..

[182] Hughes L., Black L., Sherriff J., Dunlop E., Strobel N., Lucas R., Bornman J. (2018). Vitamin D Content of Australian Native Food Plants and Australian-Grown Edible Seaweed. Nutrients.

[183] Nam P.V., Van Hoa N., Anh T.T.L., Trung T.S. (2020). Towards Zero-Waste Recovery of Bioactive Compounds from Catfish (Pangasius Hypophthalmus) By-Products Using an Enzymatic Method. Waste Biomass Valorization.

[184] Rasyid A. (2017). Evaluation of Nutritional Composition of The Dried Seaweed Ulva Lactuca from Pameungpeuk Waters, Indonesia. Trop. Life Sci. Res..

[185] Aparna P., Muthathal S., Nongkynrih B., Gupta S. (2018). Vitamin D Deficiency in India. J. Fam. Med. Prim. Care.

[186] Holick M.F. (2017). The Vitamin D Deficiency Pandemic: Approaches for Diagnosis, Treatment and Prevention. Rev. Endocr. Metab. Disord..

[187] Cashman K.D., Dowling K.G., Škrabáková Z., Gonzalez-Gross M., Valtueña J., De Henauw S., Moreno L., Damsgaard C.T., Michaelsen K.F., Mølgaard C. (2016). Vitamin D Deficiency in Europe: Pandemic?. Am. J. Clin. Nutr..

[188] Midtbø L.K., Nygaard L.B., Markhus M.W., Kjellevold M., Lie Ø., Dahl L., Kvestad I., Frøyland L., Graff I.E., Øyen J. (2020). Vitamin D Status in Preschool Children and Its Relations to Vitamin D Sources and Body Mass Index—Fish Intervention Studies-KIDS (FINS-KIDS). Nutrition.

[189] Aakre I., Næss S., Kjellevold M., Markhus M.W., Alvheim A.R., Dalane J.Ø., Kielland E., Dahl L. (2019). New Data on Nutrient Composition in Large Selection of Commercially Available Seafood Products and Its Impact on Micronutrient Intake. Food Nutr. Res..

[190] Jääskeläinen T., Itkonen S.T., Lundqvist A., Erkkola M., Koskela T., Lakkala K., Dowling K.G., Hull G.L., Kröger H., Karppinen J. (2017). The Positive Impact of General Vitamin D Food Fortification Policy on Vitamin D Status in a Representative Adult Finnish Population: Evidence from an 11-y Follow-up Based on Standardized 25-Hydroxyvitamin D Data. Am. J. Clin. Nutr..

[191] Al Khalifah R., Alsheikh R., Alnasser Y., Alsheikh R., Alhelali N., Naji A., Al Backer N. (2020). The Impact of Vitamin D Food Fortification and Health Outcomes in Children: A Systematic Review and Meta-Regression. Syst. Rev..

[192] Emadzadeh M., Sahebi R., Khedmatgozar H., Sadeghi R., Farjami M., Sharifan P., Ravanshad Y., Ferns G.A., Ghayour-Mobarhan M. (2020). A Systematic Review and Meta-analysis of the Effect of Vitamin D-fortified Food on Glycemic Indices. BioFactors.

[193] Jahn S., Tsalis G., Lähteenmäki L. (2019). How Attitude towards Food Fortification Can Lead to Purchase Intention. Appetite.

[194] Ciriminna R., Meneguzzo F., Delisi R., Pagliaro M. (2017). Enhancing and Improving the Extraction of Omega-3 from Fish Oil. Sustain. Chem. Pharm..

[195] Ciriminna R., Scurria A., Avellone G., Pagliaro M. (2019). A Circular Economy Approach to Fish Oil Extraction. ChemistrySelect.

[196] Viera I., Pérez-Gálvez A., Roca M. (2018). Bioaccessibility of Marine Carotenoids. Mar. Drugs.

[197] Tyśkiewicz K., Gieysztor R., Maziarczyk I., Hodurek P., Rój E., Skalicka-Woźniak K. (2018). Supercritical Fluid Chromatography with Photodiode Array Detection in the Determination of Fat-Soluble Vitamins in Hemp Seed Oil and Waste Fish Oil. Molecules.

[198] Azzi A. (2019). Tocopherols, Tocotrienols and Tocomonoenols: Many Similar Molecules but Only One Vitamin E. Redox Biol..

[199] Gómez-Estaca J., Calvo M.M., Álvarez-Acero I., Montero P., Gómez-Guillén M.C. (2017). Characterization and Storage Stability of Astaxanthin Esters, Fatty Acid Profile and α-Tocopherol of Lipid Extract from Shrimp (L. Vannamei) Waste with Potential Applications as Food Ingredient. Food Chem..

[200] Feng X., Tjia J.Y.Y., Zhou Y., Liu Q., Fu C., Yang H. (2020). Effects of Tocopherol Nanoemulsion Addition on Fish Sausage Properties and Fatty Acid Oxidation. LWT.

[201] Honold P.J., Nouard M.L., Jacobsen C. (2016). Fish Oil Extracted from Fish-Fillet by-Products Is Weakly Linked to the Extraction Temperatures but Strongly Linked to the Omega-3 Content of the Raw Material. Eur. J. Lipid Sci. Technol..

[202] Halder M., Petsophonsakul P., Akbulut A., Pavlic A., Bohan F., Anderson E., Maresz K., Kramann R., Schurgers L. (2019). Vitamin K: Double Bonds beyond Coagulation Insights into Differences between Vitamin K1 and K2 in Health and Disease. Int. J. Mol. Sci..

[203] Vermeer C., Raes J., van’t Hoofd C., Knapen M., Xanthoulea S. (2018). Menaquinone Content of Cheese. Nutrients.

[204] Kamao M., Suhara Y., Tsugawa N., Uwano M., Yamaguchi N., Uenishi K., Ishida H., Sasaki S., Okano T. (2007). Vitamin K Content of Foods and Dietary Vitamin K Intake in Japanese Young Women. J. Nutr. Sci. Vitaminol. (Tokyo).

[205] Tarento T.D.C., McClure D.D., Vasiljevski E., Schindeler A., Dehghani F., Kavanagh J.M. (2018). Microalgae as a Source of Vitamin K1. Algal Res..

[206] Tarento T.D.C., McClure D.D., Talbot A.M., Regtop H.L., Biffin J.R., Valtchev P., Dehghani F., Kavanagh J.M. (2019). A Potential Biotechnological Process for the Sustainable Production of Vitamin K 1. Crit. Rev. Biotechnol..

[207] Simes D.C., Viegas C.S.B., Araújo N., Marreiros C. (2020). Vitamin K as a Diet Supplement with Impact in Human Health: Current Evidence in Age-Related Diseases. Nutrients.

[208] Rizzo G., Laganà A., Rapisarda A., La Ferrera G., Buscema M., Rossetti P., Nigro A., Muscia V., Valenti G., Sapia F. (2016). Vitamin B12 among Vegetarians: Status, Assessment and Supplementation. Nutrients.

[209] Kim Y.-N., Hwang J.H., Cho Y.-O. (2018). One-Half of Korean Adults Studied Had Marginal Vitamin B 12 Status Assessed by Plasma Vitamin B 12. Nutr. Res..

[210] Marushka L., Kenny T.-A., Batal M., Cheung W.W.L., Fediuk K., Golden C.D., Salomon A.K., Sadik T., Weatherdon L.V., Chan H.M. (2019). Potential Impacts of Climate-Related Decline of Seafood Harvest on Nutritional Status of Coastal First Nations in British Columbia, Canada. PLoS ONE.

[211] Bito T., Tanioka Y., Watanabe F. (2018). Characterization of Vitamin B12 Compounds from Marine Foods. Fish. Sci..

[212] Iemolo A., De Risi M., De Leonibus E. (2015). Role of Dopamine in Memory Consolidation. Memory Consolidation.

[213] Pace-Schott E.F. (2011). The Neurobiology of Dreaming. Principles and Practice of Sleep Medicine.

[214] Perogamvros L., Dang-Vu T.T., Desseilles M., Schwartz S. (2013). Sleep and Dreaming Are for Important Matters. Front. Psychol..

[215] Tokunaga N., Choudhury M.E., Nishikawa N., Nagai M., Tujii T., Iwaki H., Kaneta M., Nomoto M. (2012). Pramipexole Upregulates Dopamine Receptor D2 and D3 Expression in Rat Striatum. J. Pharmacol. Sci..

[216] Hondebrink L., Tan S., Hermans E., van Kleef R.G.D.M., Meulenbelt J., Westerink R.H.S. (2013). Additive Inhibition of Human A1β2γ2 GABAA Receptors by Mixtures of Commonly Used Drugs of Abuse. Neurotoxicology.

[217] Szyrwiel L., Pap J.S., Malinka W., Szewczuk Z., Kotynia A., Brasun J. (2013). Interactions of Anti-Parkinson Drug Benserazide with Zn(II), Cu(II), Fe(II) Ions. J. Pharm. Biomed. Anal..

[218] Tarazi F.I., Neill J.C. (2013). The Preclinical Profile of Asenapine: Clinical Relevance for the Treatment of Schizophrenia and Bipolar Mania. Expert Opin. Drug Discov..

[219] Saikia A., Bhattacharya P., Sudip P. (2018). Importance of Dopamine in Parkinson’s Disease. Adv. Tissue Eng. Regen. Med. Open Access.

[220] Pacifici G.M. (2014). Clinical Pharmacology of Dobutamine and Dopamine in Preterm Neonates. Med. Express.

[221] Dilli D., Soylu H., Tekin N. (2019). Turkish Neonatal Society Guideline on the Neonatal Hemodynamics and Management of Hypotension in Newborns. Türk Pediatr. Arşivi.

[222] Derby C.D., Kicklighter C.E., Johnson P.M., Zhang X. (2007). Chemical Composition of Inks of Diverse Marine Molluscs Suggests Convergent Chemical Defenses. J. Chem. Ecol..

[223] Gleadall I.G., Guerrero-Kommritz J., Hochberg F.G., Laptikhovsky V.V. (2010). The Inkless Octopuses (Cephalopoda: Octopodidae) of the Southwest Atlantic. Zoolog. Sci..

[224] Derby C. (2014). Cephalopod Ink: Production, Chemistry, Functions and Applications. Mar. Drugs.

[225] Fahmy S.R., Soliman A.M., Ali E.M. (2014). Antifungal and Antihepatotoxic Effects of Sepia Ink Extract against Oxidative Stress as a Risk Factor of Invasive Pulmonary Aspergillosis in Neutropenic Mice. Afr. J. Tradit. Complement. Altern. Med..

[226] Jismi J., Krishnakumar K., Dineshkumar B. (2018). Squid Ink and Its Pharmacological Activities. GSC Biol. Pharm. Sci..

[227] Palumbo A., Di Cosmo A., Gesualdo I., Hearing V.J. (1997). Subcellular Localization and Function of Melanogenic Enzymes in the Ink Gland of Sepia Officinalis. Biochem. J..

[228] Lucero M.T., Farrington H., Gilly W.F. (1994). Quantification of L-Dopa and Dopamine in Squid Ink: Implications for Chemoreception. Biol. Bull..

[229] Fiore G., Poli A., Di Cosmo A., D’ischia M., Palumbo A. (2004). Dopamine in the Ink Defence System of Sepia Officinalis: Biosynthesis, Vesicular Compartmentation in Mature Ink Gland Cells, Nitric Oxide (NO)/CGMP-Induced Depletion and Fate in Secreted Ink1. Biochem. J..

[230] Naila A., Flint S., Fletcher G., Bremer P., Meerdink G. (2010). Control of Biogenic Amines in Food-Existing and Emerging Approaches. J. Food Sci..

[231] Bales J.W., Kline A.E., Wagner A.K., Dixon C.E. (2010). Targeting Dopamine in Acute Traumatic Brain Injury. Open Drug Discov. J..

[232] Khalid S., Abbas M., Bader-Ul-Ain H., Hafiz Ansar Rasul H., Goyal M.R., Suleria H.A.R., Kirubanandan S. (2019). Pharmacological Applications Of Marine-Derived Compounds: A Preventive Approach. Technological Processes for Marine Foods-from Water to Fork: Bioactive Compounds, Industrial Applications and Genomics.

[233] Hwang D., Kang M., Jo M., Seo Y., Park N., Kim G.-D. (2019). Anti-Inflammatory Activity of β-Thymosin Peptide Derived from Pacific Oyster (Crassostrea Gigas) on NO and PGE2 Production by Down-Regulating NF-ΚB in LPS-Induced RAW264.7 Macrophage Cells. Mar. Drugs.

[234] Scarfì S., Pozzolini M., Oliveri C., Mirata S., Salis A., Damonte G., Fenoglio D., Altosole T., Ilan M., Bertolino M. (2020). Identification, Purification and Molecular Characterization of Chondrosin, a New Protein with Anti-Tumoral Activity from the Marine Sponge Chondrosia Reniformis Nardo 1847. Mar. Drugs.

[235] Sila A., Bougatef A. (2016). Antioxidant Peptides from Marine By-Products: Isolation, Identification and Application in Food Systems. A Review. J. Funct. Foods.

[236] Nwachukwu I.D., Aluko R.E. (2019). Structural and Functional Properties of Food Protein-Derived Antioxidant Peptides. J. Food Biochem..

[237] Aluko R.E. (2015). Antihypertensive Peptides from Food Proteins. Annu. Rev. Food Sci. Technol..

[238] Abachi S., Bazinet L., Beaulieu L. (2019). Antihypertensive and Angiotensin-i-Converting Enzyme (ACE)-Inhibitory Peptides from Fish as Potential Cardioprotective Compounds. Mar. Drugs.

[239] Pujiastuti D.Y., Ghoyatul Amin M.N., Alamsjah M.A., Hsu J.L. (2019). Marine Organisms as Potential Sources of Bioactive Peptides That Inhibit the Activity of Angiotensin I-Converting Enzyme: A Review. Molecules.

[240] Kim S.K., Wijesekara I. (2010). Development and Biological Activities of Marine-Derived Bioactive Peptides: A Review. J. Funct. Foods.

[241] Byun H.-G., Lee J.K., Park H.G., Jeon J.-K., Kim S.-K. (2009). Antioxidant Peptides Isolated from the Marine Rotifer, Brachionus Rotundiformis. Process Biochem..

[242] Bougatef A., Nedjar-Arroume N., Manni L., Ravallec R., Barkia A., Guillochon D., Nasri M. (2010). Purification and Identification of Novel Antioxidant Peptides from Enzymatic Hydrolysates of Sardinelle (Sardinella Aurita) by-Products Proteins. Food Chem..

[243] Bashir K.M.I., Sohn J.H., Kim J.S., Choi J.S. (2020). Identification and Characterization of Novel Antioxidant Peptides from Mackerel (Scomber Japonicus) Muscle Protein Hydrolysates. Food Chem..

[244] Balti R., Bougatef A., Sila A., Guillochon D., Dhulster P., Nedjar-Arroume N. (2015). Nine Novel Angiotensin I-Converting Enzyme (ACE) Inhibitory Peptides from Cuttlefish (Sepia Officinalis) Muscle Protein Hydrolysates and Antihypertensive Effect of the Potent Active Peptide in Spontaneously Hypertensive Rats. Food Chem..

[245] Ko J.Y., Kang N., Lee J.H., Kim J.S., Kim W.S., Park S.J., Kim Y.T., Jeon Y.J. (2016). Angiotensin I-Converting Enzyme Inhibitory Peptides from an Enzymatic Hydrolysate of Flounder Fish (Paralichthys Olivaceus) Muscle as a Potent Anti-Hypertensive Agent. Process Biochem..

[246] Lan X., Liao D., Wu S., Wang F., Sun J., Tong Z. (2015). Rapid Purification and Characterization of Angiotensin Converting Enzyme Inhibitory Peptides from Lizard Fish Protein Hydrolysates with Magnetic Affinity Separation. Food Chem..

[247] Ngo D.-H., Vo T.-S., Ryu B., Kim S.-K. (2016). Angiotensin-I-Converting Enzyme (ACE) Inhibitory Peptides from Pacific Cod Skin Gelatin Using Ultrafiltration Membranes. Process Biochem..

[248] Kleekayai T., Harnedy P.A., O’Keeffe M.B., Poyarkov A.A., Cunhaneves A., Suntornsuk W., Fitzgerald R.J. (2015). Extraction of Antioxidant and ACE Inhibitory Peptides from Thai Traditional Fermented Shrimp Pastes. Food Chem..

[249] Liu X., Zhang M., Jia A., Zhang Y., Zhu H., Zhang C., Sun Z., Liu C. (2013). Purification and Characterization of Angiotensin I Converting Enzyme Inhibitory Peptides from Jellyfish Rhopilema Esculentum. Food Res. Int..

[250] López-Abarrategui C., Alba A., Silva O.N., Reyes-Acosta O., Vasconcelos I.M., Oliveira J.T.A., Migliolo L., Costa M.P., Costa C.R., Silva M.R.R. (2012). Functional Characterization of a Synthetic Hydrophilic Antifungal Peptide Derived from the Marine Snail Cenchritis Muricatus. Biochimie.

[251] Vo T.S., Kim S.K. (2013). Down-Regulation of Histamine-Induced Endothelial Cell Activation as Potential Anti-Atherosclerotic Activity of Peptides from Spirulina Maxima. Eur. J. Pharm. Sci..

[252] Lee H.A., Kim I.H., Nam T.J. (2015). Bioactive Peptide from Pyropia Yezoensis and Its Anti-Inflammatory Activities. Int. J. Mol. Med..

[253] Ngo D.H., Kang K.H., Ryu B., Vo T.S., Jung W.K., Byun H.G., Kim S.K. (2014). Angiotensin-I Converting Enzyme Inhibitory Peptides from Antihypertensive Skate (Okamejei Kenojei) Skin Gelatin Hydrolysate in Spontaneously Hypertensive Rats. Food Chem..

[254] Fitzgerald C., Aluko R.E., Hossain M., Rai D.K., Hayes M. (2014). Potential of a Renin Inhibitory Peptide from the Red Seaweed Palmaria Palmata as a Functional Food Ingredient Following Confirmation and Characterization of a Hypotensive Effect in Spontaneously Hypertensive Rats. J. Agric. Food Chem..

[255] Song R., Wei R.B., Luo H.Y., Yang Z.S. (2014). Isolation and Identification of an Antiproliferative Peptide Derived from Heated Products of Peptic Hydrolysates of Half-Fin Anchovy (Setipinna Taty). J. Funct. Foods.

[256] Wang M., Nie Y., Peng Y., He F., Yang J., Wu C., Li X. (2012). Purification, Characterization and Antitumor Activities of a New Protein from Syngnathus Acus, an Officinal Marine Fish. Mar. Drugs.

[257] Himaya S.W.A., Ryu B., Ngo D.H., Kim S.K. (2012). Peptide Isolated from Japanese Flounder Skin Gelatin Protects against Cellular Oxidative Damage. J. Agric. Food Chem..

[258] Indumathi P., Mehta A. (2016). A Novel Anticoagulant Peptide from the Nori Hydrolysate. J. Funct. Foods.

[259] Fan X., Bai L., Mao X., Zhang X. (2017). Novel Peptides with Anti-Proliferation Activity from the Porphyra Haitanesis Hydrolysate. Process Biochem..

[260] Harnedy P.A., O’Keeffe M.B., FitzGerald R.J. (2017). Fractionation and Identification of Antioxidant Peptides from an Enzymatically Hydrolysed Palmaria Palmata Protein Isolate. Food Res. Int..

[261] Admassu H., Gasmalla M.A.A., Yang R., Zhao W. (2018). Identification of Bioactive Peptides with α-Amylase Inhibitory Potential from Enzymatic Protein Hydrolysates of Red Seaweed (Porphyra Spp). J. Agric. Food Chem..

[262] Wang K., Siddanakoppalu P.N., Ahmed I., Pavase T.R., Lin H., Li Z. (2020). Purification and Identification of Anti-Allergic Peptide from Atlantic Salmon (Salmo Salar) Byproduct Enzymatic Hydrolysates. J. Funct. Foods.

[263] Najafian L., Babji A.S. (2018). Purification and Identification of Antioxidant Peptides from Fermented Fish Sauce (Budu) Purification and Identification of Antioxidant Peptides From. J. Aquat. Food Prod. Technol..

[264] Yang X.R., Qiu Y.T., Zhao Y.Q., Chi C.F., Wang B. (2019). Purification and Characterization of Antioxidant Peptides Derived from Protein Hydrolysate of the Marine Bivalve Mollusk Tergillarca Granosa. Mar. Drugs.

[265] Aissaoui N., Abidi F., Hardouin J., Abdelkafi Z., Marrakchi N., Jouenne T., Marzouki M.N. (2017). ACE Inhibitory and Antioxidant Activities of Novel Peptides from Scorpaena Notata By-Product Protein Hydrolysate. Int. J. Pept. Res. Ther..

[266] Liu P., Lan X., Yaseen M., Wu S., Feng X., Zhou L., Sun J., Liao A., Liao D., Sun L. (2019). Purification, Characterization and Evaluation of Inhibitory Mechanism of ACE Inhibitory Peptides from Pearl Oyster (Pinctada Fucata Martensii) Meat Protein Hydrolysate. Mar. Drugs.

[267] Tao J., Zhao Y.Q., Chi C.F., Wang B. (2018). Bioactive Peptides from Cartilage Protein Hydrolysate of Spotless Smoothhound and Their Antioxidant Activity In Vitro. Mar. Drugs.

[268] Montone C.M., Capriotti A.L., Cavaliere C., La Barbera G., Piovesana S., Zenezini Chiozzi R., Laganà A. (2018). Peptidomic Strategy for Purification and Identification of Potential ACE-Inhibitory and Antioxidant Peptides in Tetradesmus Obliquus Microalgae. Anal. Bioanal. Chem..

[269] Narayana J.L., Huang H.N., Wu C.J., Chen J.Y. (2015). Efficacy of the Antimicrobial Peptide TP4 against Helicobacter Pylori Infection: In Vitro Membrane Perturbation via Micellization and in Vivo Suppression of Host Immune Responses in a Mouse Model. Oncotarget.

[270] Zhong C., Sun L.C., Yan L.J., Lin Y.C., Liu G.M., Cao M.J. (2018). Production, Optimisation and Characterisation of Angiotensin Converting Enzyme Inhibitory Peptides from Sea Cucumber (: Stichopus Japonicus) Gonad. Food Funct..

[271] Quah Y., Mohd Ismail N.I., Ooi J.L.S., Affendi Y.A., Abd Manan F., Wong F.C., Chai T.T. (2018). Identification of Novel Cytotoxic Peptide KENPVLSLVNGMF from Marine Sponge Xestospongia Testudinaria, with Characterization of Stability in Human Serum. Int. J. Pept. Res. Ther..

[272] Lv L.C., Huang Q.Y., Ding W., Xiao X.H., Zhang H.Y., Xiong L.X. (2019). Fish Gelatin: The Novel Potential Applications. J. Funct. Foods.

[273] Shahidi F., Varatharajan V., Peng H., Senadheera R. (2019). Utilization of Marine By-Products for the Recovery of Value-Added Products. J. Food Bioact..

[274] Tkaczewska J., Morawska M., Kulawik P., Zając M. (2018). Characterization of Carp (Cyprinus Carpio) Skin Gelatin Extracted Using Different Pretreatments Method. Food Hydrocoll..

[275] Bello A.B., Kim D., Kim D., Park H., Lee S.H. (2020). Engineering and Functionalization of Gelatin Biomaterials: From Cell Culture to Medical Applications. Tissue Eng. Part B Rev..

[276] Plazzotta S., Manzocco L. (2019). Food Waste Valorization.

[277] Xu M., Wei L., Xiao Y., Bi H., Yang H., Du Y. (2017). Physicochemical and Functional Properties of Gelatin Extracted from Yak Skin. Int. J. Biol. Macromol..

[278] Bhat R., Karim A.A. (2009). Ultraviolet Radiation Improves Gel Strength of Fish Gelatin. Food Chem..

[279] Kwak H.W., Shin M., Lee J.Y., Yun H., Song D.W., Yang Y., Shin B.-S., Park Y.H., Lee K.H. (2017). Fabrication of an Ultrafine Fish Gelatin Nanofibrous Web from an Aqueous Solution by Electrospinning. Int. J. Biol. Macromol..

[280] Aksun Tumerkan E.T., Cansu U., Boran G., Regenstein J.M., Ozogul F. (2019). Physiochemical and Functional Properties of Gelatin Obtained from Tuna, Frog and Chicken Skins. Food Chem..

[281] Sharif R. (2019). Gelatin; Switch Back to Halal: A Mini-Review. PSM Biol. Res..

[282] Karaman S., Cengiz E., Kayacier A., Dogan M. (2016). Exposure to Air Accelerates the Gelation of Gelatin: Steady and Dynamic Shear Rheological Characterization to See the Effect of Air on the Strength of Gelatin Gel. Int. J. Food Prop..

[283] Huang T., Tu Z.C., Wang H., Liu W., Zhang L., Zhang Y., ShangGuan X.C. (2017). Comparison of Rheological Behaviors and Nanostructure of Bighead Carp Scales Gelatin Modified by Different Modification Methods. J. Food Sci. Technol..

[284] You L., Zhao M., Regenstein J.M., Ren J. (2010). Purification and Identification of Antioxidative Peptides from Loach (Misgurnus Anguillicaudatus) Protein Hydrolysate by Consecutive Chromatography and Electrospray Ionization-Mass Spectrometry. Food Res. Int..

[285] Koli J.M., Basu S., Nayak B.B., Nagalakshmi K., Venkateshwarlu G. (2011). Improvement of Gel Strength and Melting Point of Fish Gelatin by Addition of Coenhancers Using Response Surface Methodology. J. Food Sci..

[286] Jridi M., Souissi N., Mbarek A., Chadeyron G., Kammoun M., Nasri M. (2013). Comparative Study of Physico-Mechanical and Antioxidant Properties of Edible Gelatin Films from the Skin of Cuttlefish. Int. J. Biol. Macromol..

[287] Jeevithan E., Qingbo Z., Bao B., Wu W. (2013). Biomedical and Pharmaceutical Application of Fish Collagen and Gelatin: A Review. J. Nutr. Ther..

[288] Loo C.P.Y., Sarbon N.M. (2020). Chicken Skin Gelatin Films with Tapioca Starch. Food Biosci..

[289] Manikandan A., Thirupathi Kumara Raja S., Thiruselvi T., Gnanamani A. (2018). Engineered Fish Scale Gelatin: An Alternative and Suitable Biomaterial for Tissue Engineering. J. Bioact. Compat. Polym..

[290] Mahmoudi Saber M. (2019). Strategies for Surface Modification of Gelatin-Based Nanoparticles. Colloids Surfaces B Biointerfaces.

[291] Qureshi D., Nayak S.K., Anis A., Ray S.S., Kim D., Hanh Nguyen T.T., Pal K. (2020). Introduction of Biopolymers.

[292] Du C., Abdullah J.J., Greetham D., Fu D., Yu M., Ren L., Li S., Lu D. (2018). Valorization of Food Waste into Biofertiliser and Its Field Application. J. Clean. Prod..

[293] Weiss A.V., Fischer T., Iturri J., Benitez R., Toca-Herrera J.L., Schneider M. (2019). Mechanical Properties of Gelatin Nanoparticles in Dependency of Crosslinking Time and Storage. Colloids Surfaces B Biointerfaces.

[294] Ceylan Z., Unal SengOr G.F., Yilmaz M.T. (2017). Amino Acid Composition of Gilthead Sea Bream Fillets (Sparus Aurata) Coated with Thymol-Loaded Chitosan Nanofibers during Cold Storage. J. Biotechnol..

[295] Ceylan Z., Meral R., Cavidoglu I., Yagmur Karakas C., Tahsin Yilmaz M. (2018). A New Application on Fatty Acid Stability of Fish Fillets: Coating with Probiotic Bacteria-loaded Polymer-based Characterized Nanofibers. J. Food Saf..

[296] Ceylan Z., Yaman M., Sağdıç O., Karabulut E., Yilmaz M.T. (2018). Effect of Electrospun Thymol-Loaded Nanofiber Coating on Vitamin B Profile of Gilthead Sea Bream Fillets (Sparus Aurata). LWT.

[297] Swanson D., Block R., Mousa S.A. (2012). Omega-3 Fatty Acids EPA and DHA: Health Benefits Throughout Life. Adv. Nutr..

[298] Ceylan Z., Meral R., Karakaş C.Y., Dertli E., Yilmaz M.T. (2018). A Novel Strategy for Probiotic Bacteria: Ensuring Microbial Stability of Fish Fillets Using Characterized Probiotic Bacteria-Loaded Nanofibers. Innov. Food Sci. Emerg. Technol..

[299] Dawczynski C., Schubert R., Jahreis G. (2007). Amino Acids, Fatty Acids, and Dietary Fibre in Edible Seaweed Products. Food Chem..

[300] Venkatraman A., Yahoob S.A.M., Nagarajan Y., Harikrishnan S., Vasudevan S., Murugasamy T. (2018). Pharmacological Activity of Biosynthesized Gold Nanoparticles from Brown Algae-Seaweed Turbinaria Conoides. NanoWorld J..

[301] Zhong J., Yang R., Cao X., Xiong Liu X., Qin X. (2018). Improved Physicochemical Properties of Yogurt Fortified with Fish Oil/γ-Oryzanol by Nanoemulsion Technology. Molecules.

[302] Chanthini A.B., Balasubramani G., Ramkumar R., Sowmiya R., Balakumaran M.D., Kalaichelvan P.T., Perumal P. (2015). Structural Characterization, Antioxidant and in Vitro Cytotoxic Properties of Seagrass, Cymodocea Serrulata (R.Br.) Asch. & Magnus Mediated Silver Nanoparticles. J. Photochem. Photobiol. B Biol..

[303] Anand B.G., Thomas C.K.N., Prakash S., Kumar C.S. (2015). Biosynthesis of Silver Nano-Particles by Marine Sediment Fungi for a Dose Dependent Cytotoxicity against HEp2 Cell Lines. Biocatal. Agric. Biotechnol..

[304] Muthuirulappan S., Francis S.P. (2013). Anti-Cancer Mechanism and Possibility of Nano-Suspension Formulation for a Marine Algae Product Fucoxanthin. Asian Pacific J. Cancer Prev..

[305] Phleger C.F., Nelson M.M., Mooney B.D., Nichols P.D. (2002). Interannual and between Species Comparison of the Lipids, Fatty Acids and Sterols of Antarctic Krill from the US AMLR Elephant Island Survey Area. Comp. Biochem. Physiol. Part B Biochem. Mol. Biol..

[306] Tou J.C., Jaczynski J., Chen Y.-C. (2008). Krill for Human Consumption: Nutritional Value and Potential Health Benefits. Nutr. Rev..

[307] Racine R.A., Deckelbaum R.J. (2007). Sources of the Very-Long-Chain Unsaturated Omega-3 Fatty Acids: Eicosapentaenoic Acid and Docosahexaenoic Acid. Curr. Opin. Clin. Nutr. Metab. Care.

[308] Haider J., Majeed H., Williams P.A., Safdar W., Zhong F. (2017). Formation of Chitosan Nanoparticles to Encapsulate Krill Oil (*Euphausia superba*) for Application as a Dietary Supplement. Food Hydrocoll..

[309] Zhou L., Yang F., Zhang M., Liu J. (2020). A Green Enzymatic Extraction Optimization and Oxidative Stability of Krill Oil from *Euphausia superba*. Mar. Drugs.

